# Schizophrenia-associated changes in neuronal subpopulations in the human midbrain

**DOI:** 10.1093/brain/awae321

**Published:** 2024-10-14

**Authors:** Astrid M Alsema, Sofía Puvogel, Laura Kracht, Maree J Webster, Cynthia Shannon Weickert, Bart J L Eggen, Iris E C Sommer

**Affiliations:** Department of Biomedical Sciences, Section Molecular Neurobiology, University of Groningen, University Medical Center Groningen, Groningen 9713 AV, The Netherlands; Department of Biomedical Sciences, Section Molecular Neurobiology, University of Groningen, University Medical Center Groningen, Groningen 9713 AV, The Netherlands; Department of Biomedical Sciences, Section Cognitive Neuroscience, University of Groningen, University Medical Center Groningen, Groningen 9713 AW, The Netherlands; Institute of Molecular Biotechnology of the Austrian Academy of Sciences (IMBA), Vienna BioCenter (VBC), Vienna 1030, Austria; Laboratory of Brain Research, Stanley Medical Research Institute, Rockville, MD 20850, USA; Schizophrenia Research Laboratory, Neuroscience Research Australia, Sydney, NSW 2031, Australia; School of Psychiatry, University of New South Wales, Sydney, NSW 2033, Australia; Department of Neuroscience and Physiology, Upstate Medical University, Syracuse, NY 13210, USA; Department of Biomedical Sciences, Section Molecular Neurobiology, University of Groningen, University Medical Center Groningen, Groningen 9713 AV, The Netherlands; Department of Biomedical Sciences, Section Cognitive Neuroscience, University of Groningen, University Medical Center Groningen, Groningen 9713 AW, The Netherlands

**Keywords:** schizophrenia, midbrain, neurons, single-nucleus RNA sequencing

## Abstract

Dysfunctional GABAergic and dopaminergic neurons are thought to exist in the ventral midbrain of patients with schizophrenia, yet transcriptional changes underpinning these abnormalities have not yet been localized to specific neuronal subsets. In the ventral midbrain, control over dopaminergic activity is maintained by both excitatory (glutamate) and inhibitory (GABA) input neurons. To elucidate neuron pathology at the single-cell level, we characterized the transcriptional diversity of distinct NEUN+ populations in the human ventral midbrain and then tested for schizophrenia-associated changes in neuronal subset proportions and gene activity changes within neuronal subsets.

Combining single nucleus RNA-sequencing with fluorescence-activated sorting of NEUN+ nuclei, we analysed 31 669 nuclei. Initially, we detected 18 transcriptionally distinct neuronal populations in the human ventral midbrain, including two ‘mixed’ populations. The presence of neuronal populations in the midbrain was orthogonally validated with immunohistochemical stainings. ‘Mixed’ populations contained nuclei expressing transcripts for vesicular glutamate transporter 2 (*SLC17A6*) and glutamate decarboxylase 2 (*GAD2*), but these transcripts were not typically co-expressed by the same nucleus. Upon more fine-grained subclustering of the two ‘mixed’ populations, 16 additional subpopulations were identified that were transcriptionally classified as excitatory or inhibitory. In the midbrains of individuals with schizophrenia, we observed potential differences in the proportions of two (sub)populations of excitatory neurons, two subpopulations of inhibitory neurons, one ‘mixed’ subpopulation, and one subpopulation of *TH*-expressing neurons.

This may suggest that transcriptional changes associated with schizophrenia broadly affect excitatory, inhibitory, and dopamine neurons. We detected 99 genes differentially expressed in schizophrenia compared to controls within neuronal subpopulations identified from the two ‘mixed’ populations, with most (67) changes within small GABAergic neuronal subpopulations. Overall, single-nucleus transcriptomic analyses profiled a high diversity of GABAergic neurons in the human ventral midbrain, identified putative shifts in the proportion of neuronal subpopulations, and suggested dysfunction of specific GABAergic subpopulations in schizophrenia, providing directions for future research.

## Teaser

Using single-nucleus transcriptional profiling of neurons from the ventral midbrain, Alsema *et al.* identify differences in the relative abundance of various neuronal populations between individuals with and without schizophrenia. Schizophrenia is mostly associated with gene expression changes within specific GABAergic subpopulations.

## Introduction

Schizophrenia is a psychiatric disorder with heterogeneous symptoms that can lead to a lifetime of disability.^1^ Treatment is symptomatic, as the pathophysiology of schizophrenia remains largely unknown. This emphasizes the need to decipher schizophrenia pathophysiology at the molecular level so that more effective treatments can be developed targeting underlying mechanisms of the disorder.

For that purpose, large-scale RNA sequencing (RNA-seq) and co-expression networks have aided the identification of neuronal and immune pathways that are dysregulated in schizophrenia.^2,3^ Schizophrenia susceptibility genes are involved in synaptic differentiation, synaptic signalling and pre- and postsynaptic organization.^4^ Genetic and transcriptional evidence indicates that synapses of both cortical and subcortical neurons are affected, including pyramidal neurons, medium spiny neurons and interneurons.^4,5^ These studies could be expanded by considering neuronal cell populations from the adult human midbrain, a region outside the telencephalon but strongly implicated in the pathophysiology of schizophrenia.

The human ventral midbrain, which can roughly be divided into the ventral tegmental area (VTA), substantia nigra pars compacta (SNpc) and substantia nigra pars reticulum (SNpr), is a complex region^6^ where multiple neurotransmitter systems involved in schizophrenia pathology converge. Functional MRI in unmedicated schizophrenia patients indicated reduced midbrain connectivity compared to controls.^7^ Abnormalities in the midbrain, especially of dopamine-associated activity, have been demonstrated during reward prediction tasks, which shows more error in those developing psychosis.^8,9^

Animal models for schizophrenia indicate that increased dopamine firing in the midbrain may result from the loss of GABAergic interneurons in the hippocampus and increased excitatory input into the midbrain.^10-12^ Local control of midbrain dopaminergic (DA) neurons is coordinated by various GABAergic interneurons and co-laterals of GABAergic projection neurons.^13,14^ Reduced gene expression of *GAD1*, *SLC32A1* (encoding the vesicular GABA transporter VGAT), *PVALB* and *GABRA1*, *-2*, *-3*, *-5* (encoding subunits of the GABAA receptor) has been reported in the midbrain of patients with schizophrenia.^15^ GABAergic neurons have therefore been hypothesized to play a crucial role in schizophrenia pathophysiology. Furthermore, increased glutamate release may also affect DA activity and reward behaviours, as glutamatergic neurons establish local excitatory connections with neighbouring DA neurons^16,17^ and project to reward system-related brain regions.^18,19^ Until now, the exact population of neurons involved in schizophrenia and the molecular mechanism behind putative blunted midbrain GABAergic inhibition and/or increased glutamatergic excitation remains to be determined. Single nucleus profiling has identified at least five distinct neuronal subpopulations in the human midbrain^20^ and at least 11 in the rat midbrain,^21^ offering the opportunity to test if these subpopulations are transcriptionally altered in schizophrenia.

In this study, we characterize 31 669 individual glutamatergic and GABAergic neuronal transcriptional profiles by combining single nucleus RNA-seq (snRNAseq) with fluorescence-activated sorting of NEUN+ nuclei from 28 midbrain samples (14 individuals with schizophrenia and 14 controls). The combination of a demographically matched cohort and the evaluation of a high total number of NEUN+ nuclei enabled the detection of low abundant neuronal subpopulations. As the human midbrain is largely *terra incognita* regarding the transcriptional landscape at cellular resolution, we first describe the neuronal populations observed in the ventral midbrain. These were validated at the protein level with immunohistochemical stainings. Second, we determine if there is an altered relative abundance of neuronal populations in the midbrain of individuals with schizophrenia compared to controls. Third, we investigate transcriptional changes within neuronal populations in the midbrain in schizophrenia compared to controls.

## Materials and methods

### Human brain tissue

Midbrain samples from 15 schizophrenia and 14 control cases were obtained from the Stanley Medical Research Institute (SMRI) Array Collection as described by Puvogel *et al.*^22^ (Supplementary Table 1). Ethical approval for the brain collection was through the Uniformed Services University for Health Sciences. Post-mortem brains were obtained from Medical Examiners with permission from the next-of-kin. After review of all medical records and interviews with the family members, at least two senior psychiatrists independently made a psychiatric diagnosis (Diagnostic and Statistical Manual of Mental Disorders IV). For the same cohort, non-neuronal blood–brain barrier cells have been characterized, and these cell types are not broadly affected in schizophrenia.^22^

### Immunohistochemical stainings

Fresh frozen 14 μm tissue sections from five unaffected control cases were double labelled with the antibody of interest (DLX1, ETV5 or CYP26B1) and with NeuN or GFAP. Briefly, sections were thawed [room temperature (RT), 30 min] and fixed in 4% paraformaldehyde in PBS for 10 min at 40°C. Slides were treated with 75%MeOH + 0.75% H_2_O_2_ solution (RT, 20 min) and incubated with 10% normal goat serum in diluent (0.05% bovine serum albumin, 0.3% Triton X-100 in PBS) for 30 min at RT. Primary antibodies (mouse anti-DLX, 1:250, ab236381 Abcam; rabbit anti-ETV5, 1:1500, HPA073889 Millipore; rabbit anti-CYP26B1,1:400, HPA012567 Millipore) were applied overnight at 40°C, followed by appropriate secondary antibody, anti-mouse (1:100) or anti-rabbit IgG biotinylated secondary antibody (1:100, Vector Laboratories) for 1 h at RT. Sections were then incubated at RT in avidin-biotin-peroxidase complex (Vectastain ABC kit, mouse PK 4002 or rabbit PK-4001, Vector Laboratories) for 1 h and treated with DAB (Sigma; 12 nM in PBS with 0.003% H_2_O_2_) for 5–7 min. Slides were then rinsed and blocked with serum (30 min) and incubated with anti-glial fibrillary acidic protein (GFAP) primary antibody (1:500, Abcam, ab68438) at 40°C overnight and anti-rabbit secondary antibody (1:500, Vector Laboratories) at RT for 1 h. Slides were then rinsed and incubated with Vector SG kit (Vector Laboratories, SK-4700) before rinsing and coverslipping.

### Tissue selection

**Figure 1:**
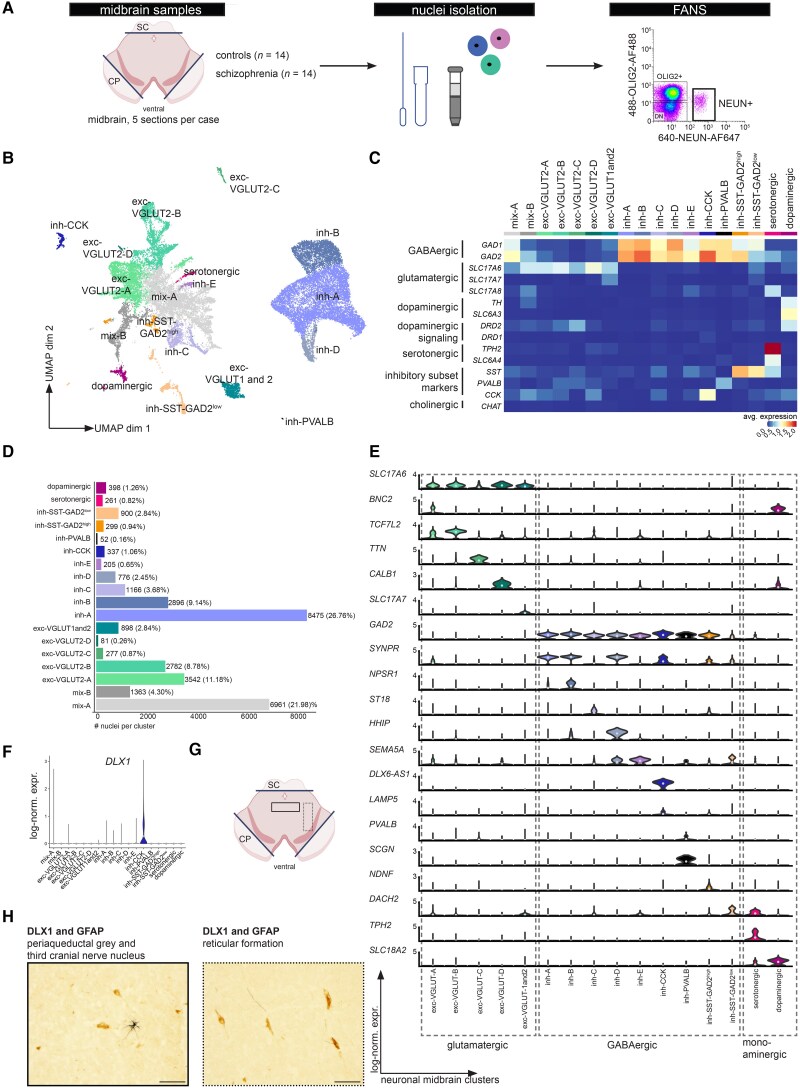
**Unsupervised clustering identifies 18 neuronal populations in the human midbrain**. (**A**) Experimental design of the study (Supplementary Table 1). A schematic representation of the rostral midbrain at the level of the red nucleus and third cranial nerves (created with biorender.com). The lines indicate where the superior colliculus (SC) and cerebral peduncles (CP) were removed before nuclei isolation, FANS and snRNAseq. (**B**) Unsupervised clustering of 31 669 neuronal nuclei from 14 schizophrenia (SZ) cases and 14 controls (Supplementary Tables 2 and 3). Each dot indicates a nucleus, colours indicate clusters. (**C**) Average expression per cluster of selected neurotransmitter-, neuropeptide- or interneuron-related genes. (**D**) Bar plot depicting the total number of nuclei obtained per population, with the percentage of the total NEUN+ population enclosed in parentheses. (**E**) Violin plots depicting log-normalized expression levels of cluster markers (log_2_ fold-change *>* 0.25, *P*-adjusted *<* 0.05) (Supplementary Table 3). (**F**) Violin plot depicting log-normalized expression level of *DLX1*, a cluster marker for inh-CCK. (**G**) A schematic representation of the rostral midbrain indicating the periaqueductal grey and the third cranial nerve nucleus with a box and the reticular formation with a dotted box. In these regions the immunohistochemical staings, shown in **H**, were performed. (**H**) Representative images of immunohistochemical stainings for DLX1 (brown) and GFAP (black) in control midbrain (*n* = 5). Scale bar = 50 μm. avg. = average; expr. = expression; FANS = fluorescence-activated nuclear sorting; log-norm. = log-normalized; snRNAseq = single nucleus RNA-sequencing; UMAP = Uniform Manifold Approximation and Projection.

Five 100 μm sections were used from each frozen midbrain block. The midbrain was sectioned with alternating thick (100 μm) and thin sections (14 μm). Thin sections were used for anti-TH staining to map the extent of the SN and VTA regions. Thick sections in between TH-immunoreactive mirror sections were used in the snRNAseq experiment. The peduncles and colliculi were removed from thick sections (100 μm), retaining the ventral tegmental area and the substantia nigra (Fig. 1A). To ensure the quality of the brain tissue, RNA was isolated from the trimmed tissue area using a RNeasy Lipid Tissue mini kit (Qiagen, 74804) to determine the RNA integrity number (RIN). The RNA concentration and integrity were measured on a Bioanalyzer 2100 (Aligent). The mean RIN value of the included samples was 7.4 ± 1.2, and all of them presented a RIN value > 4 (Supplementary Table 1).

### Nuclei isolation

Nuclei were isolated from five thick (100 μm) midbrain sections per case as described by Gerrits *et al.*^23^ Briefly, after sucrose density centrifugation, nuclei were incubated with fluorescently-conjugated antibodies directed against neuronal marker NEUN (RBFOX3/NEUN 1B7 AF647 mouse mAB, Novus Biologicals, NBP1-92693AF647) and the transcription factor OLIG2 for the oligodendrocyte lineage (Anti-OLIG2 clone 211F1.1 AF488 mouse mAB, Merck Millipore, MABN50A4). Per sample, we sorted DAPI^pos^NEUN^neg^OLIG2^neg^ (double negative nuclei) and DAPI^pos^NEUN^pos^OLIG2^neg^ nuclei (neuronal nuclei) for snRNAseq. The ratio of sorted and sequenced double negative to neuronal nuclei was set to approximately 6:1 (Supplementary Table 1).

### Single nucleus RNA-sequencing library construction and sequencing

Single nucleus cDNA libraries were constructed according to the user guide of Chromium Single Cell 3′ Reagents Kit v3 (10× Genomics). All samples were pooled in equimolar ratios and sequenced on a NextSeq 500 at GenomeScan B.V. in Leiden and the Research Sequencing Facility of the UMCG, Groningen, The Netherlands. The median sequencing depth was 210 million reads per sample and the median counts per nuclei was 2714. Authors were blinded to experimental groups during sample processing, but not during data analysis.

### Single nucleus RNA-sequencing data processing

Sequencing reads were processed and aligned to the GRCh38 human genome using CellRanger (v3.0.1). Barcode filtering was performed with Abacus^24^ to distinguish barcodes containing nuclear RNA from cytoplasmic and ambient RNA. Barcodes with low intronic read counts reflect cytoplasmic or ambient RNA. The following thresholds were set to keep high-quality nuclei and remove cellular debris: (i) >100 exonic reads; (ii) >200 intronic reads; and (iii) intronic reads >exonic reads. The counts corresponding to barcodes passing all quality filters were extracted from the raw count matrix generated by Cell Ranger and loaded in R with Seurat v4.0. Nuclei with mitochondrial content >5% were removed. Integration of normalized counts from all cases was performed according to the guidelines for fast integration with reciprocal principal component analysis (rPCA) in Seurat (v4.0).^25^ Scrublet (v0.2.1) was used to remove doublets. In addition, one cluster of nuclei expressing both astrocytic and microglia marker genes was excluded due to the high chance of containing doublets. Unbiased clustering analysis followed by the examination of the expression of marker genes was used to identify all the major brain cell types.

### Single nucleus RNA-sequencing analysis of neuronal nuclei

#### Clustering

From the complete dataset, we extracted clusters expressing *RBFOX3*, *SNAP25* and *MAP2*-expression. During quality control of neuronal nuclei, schizophrenia Case 29 was excluded, because it had a low detection rate of inhibitory neurons compared to other midbrain samples (Supplementary Table 1), resulting in 14 schizophrenia and 14 control cases for downstream analysis. Data from neuronal nuclei were normalized and integrated again using canonical correlation analysis. Uninteresting sources of variation associated with the number of unique molecular identifiers (UMIs), number of detected genes and mitochondrial content were regressed out. We removed multiple clusters representing doublets (e.g. microglia-neuron doublet, astrocyte-neuron doublet clusters), clusters with low UMI content and a cluster with high ribosomal content. After quality control, on average 1131 neuronal nuclei per case were analysed. Unbiased clustering was performed using the Seurat workflow with clustering parameters dims = 1:50, k.param = 20 and resolution = 0.2 to identify the neuronal populations.

#### Projection of clusters to published atlas on neuronal transcriptomic diversity

The previously published snRNAseq data characterizing neuronal transcriptomic diversity were obtained from https://github.com/linnarsson-lab/adult-human-brain, with the corresponding supercluster annotations published by Siletti *et al.*^26^ Data were processed to computationally select neurons from the same brain region, using the criteria ‘ROIGroupFine = midbrain’. Data annotated with ‘Dissection’ = ‘inferior colliculus and nearby nuclei—IC’ and ‘superior colliculus and nearby nuclei—SC’ were excluded from analysis, as these regions were not part of our midbrain samples. Superclusters consisting of less than 73 nuclei (<0.1% of the data) were excluded, resulting in 11 superclusters. The snRNAseq data were analysed with the default Seurat (v4.0) workflow to obtain PCA and Uniform Manifold Approximation and Projection (UMAP) representations with parameters ‘npcs’ and ‘dims’ set to 50 dimensions. To project midbrain clusters (reference) onto the published atlas data (query) using the PCA structure, we used the Seurat functions FindTransferAnchors() and TransferData() with dims = 1:50. The resulting prediction scores were visualized in UMAP representations. To set the predicted nucleus identity, nuclei with a low-confidence prediction score < 0.59 (corresponding to the 10th percentile of the prediction score distribution) were disregarded, and all further nuclei identities were set to the cluster with the maximum prediction score.

#### Co-expression of two genes

Co-expression of two genes was computed using a custom script, as the default Seurat function FeaturePlot(blend = TRUE) did not clearly visualize lowly expressed genes, such as *PVALB* and *SLC17A7*. A gene was considered expressed if its expression in a nucleus exceeded a defined threshold, which was set as the 50th percentile of the gene’s count distribution.

#### Subclustering

Subclustering for DA neurons was performed by extracting the nuclei that co-expressed *TH* and *SLC6A3*. The normalization and Seurat clustering workflow was repeated for the DA neuronal nuclei with the clustering parameters dims = 1:50, k.param = 20 and resolution = 0.5. Subclustering of mixes was performed by extracting nuclei belonging to mix-A and mix-B clusters and rerunning the clustering workflow. Four clusters with low UMI content were detected and excluded in additional quality control steps. After quality control, unsupervised clustering was performed with the parameters dims = 1:50, k.param = 20 and resolution = 0.3.

#### Gene ontology enrichment analysis

Gene ontology analysis was performed on significant cluster markers (log_2_ fold-change > 0.25, adjusted *P-*value < 0.05) and schizophrenia-associated genes using the function *compareCluster* from the clusterProfiler (v4.4.4). The arguments used were ont = BP, OrgDb = org.Hs.eg.db and keyType = symbol.

#### Genome-wide association study

The 302 high-confidence genes corresponding to schizophrenia susceptibility genes were obtained from a meta-analysis by Wang *et al.*^27^ available at http://resource.psychencode.org/ under the filename ‘INT-17_SCZ_High_confidence_gene_list’ as well as in Supplementary Table 15. Mapping the expression of previously prioritized genome-wide association study (GWAS) susceptibility was conducted according to the procedure reported by Absinta *et al.*^28^ Briefly, genes that are lowly expressed in single-nucleus RNAseq data (lower than the 25th percentile) were excluded, resulting in 213 high-confidence schizophrenia susceptibility genes. Gene expression was averaged per population and scaled, allowing comparison of relative expression between populations. Genes were defined as detected in a population if the z-score of average gene expression was >1.5. The normalized ratio was computed by dividing the number of detected schizophrenia susceptibility genes by the total number of detected genes in a cluster. Overlap of gene sets was statistically assessed using the R-package GeneOverlap (v1.26.0) and GO term enrichment was assessed using the online web tool metascape.org. Venn diagrams were created by intersecting the 302 high-confidence schizophrenia susceptibility genes by Wang *et al.*^27^ with the differentially expressed genes (DEGs) altered in schizophrenia within populations (clusters) and subpopulations (subclusters).

### Statistical analysis

For more than 20 comparisons, multiple testing was controlled for by using the function *p.adjust* with the method set to ‘fdr’. For instances with fewer than 20 comparisons, the Benjamini-Hochberg procedure was computed manually.^29^

#### Differentially expressed cluster markers

Differentially expressed genes per cluster were computed with Seurat’s *FindAllMarkers* function with the parameters only.pos = FALSE, test.use = ‘MAST’ and latent.vars = ‘donor_ID’. Cluster markers were defined as genes with a log2 fold-change > 0.25 and adjusted *P-*value < 0.05 as provided by Seurat.

#### Comparing (sub)cluster proportions between schizophrenia and control samples

As described previously,^22,30^ we fitted a generalized linear model with a quasibinomial distribution to test if diagnosis (schizophrenia or control) affects the probability of a nucleus belonging to a given cluster. Afterwards, we manually corrected the obtained results for false discoveries using a false discovery rate (FDR) threshold of 0.2.^29^

#### Differential abundance testing of (sub)clusters between schizophrenia and control samples

To assess the differential abundance of neuronal (sub)clusters, we employed scCODA (v0.1.9), which uses a Bayesian framework for joint modelling of all measured cluster proportions,^31^ as opposed to treating them individually. For each test, the FDR threshold was adjusted to 0.2 and automatic reference selection was applied.

#### Differentially expressed genes between schizophrenia and control samples

To test for schizophrenia-associated transcriptional changes per (sub)cluster, the function *zlm* from the R package MAST (v1.16.0) was used.^32^ Donor-related data structure was accounted for by including a random intercept per case and this extension to mixed models prevents pseudo-replication.^33^ The cellular detection rate was included as a covariate, according to the recommendations of Finak *et al.*^32^ On the gene level, we made a prior selection of genes per (sub)cluster that meet the criteria described in Puvogel *et al.*^22^ On the sample level, we only considered samples that contributed more than 10 nuclei to the given (sub)cluster. On the (sub)cluster level, we assessed (sub)clusters that were composed of at least seven samples meeting the criteria above. Mix-A and mix-B were excluded as these populations are analysed in detail as subpopulations. Genes were identified as differentially expressed with an absolute log_2_ fold-change > 0.25 and an adjusted *P*-value < 0.05, as determined by MAST.

## Results

### Single-nucleus RNA sequencing of NEUN+ populations in the human midbrain

RBFOX3/NEUN+ nuclei were purified from post-mortem midbrain sections using fluorescence-activated nuclear sorting (FANS) (Fig. 1A). Isolated nuclei from 14 patients with schizophrenia and 14 controls were subjected to snRNAseq (see Supplementary Table 1 for demographics and sequencing metrics). After quality control, snRNAseq yielded a total of 31 669 control and schizophrenia neuronal nuclei, profiled at a median depth of 12 575 UMIs per nucleus and 4418 unique genes per nucleus (Fig. 1A and Supplementary Table 1), indicating a high-quality dataset with adequate read depth.

Eighteen distinct neuronal clusters were identified through unsupervised clustering of all the snRNAseq profiles of control and schizophrenia brains (Fig. 1B). Clusters were annotated based on the average expression of distinct neurotransmitter-specific transcripts, including solute carrier family 17 members 6, -7, -8 also known as vesicular glutamate transporters 2, -1, -3 (*SLC17A6*/VGLUT2, *SLC17A7*/VGLUT1*, SLC17A8*/VGLUT3), glutamate decarboxylase 1 and 2 (*GAD1*, *GAD2*), solute carrier family 6 member 3 (*SLC6A3*/DAT1), tryptophan hydroxylase (*TPH2*), solute carrier family 6 member 4 (*SLC6A4*/SERT1) and choline *O*-acetyltransferase (*CHAT*). In addition, transcripts related to DA signalling [tyrosine hydroxylase (*TH*), dopamine receptor D1 and 2 (*DRD1*, *DRD2*), as well as neuropeptides somatostatin (*SST*), cholecystokinin (*CCK*) and interneuron marker parvalbumin (*PVALB*)] were used for cluster annotation (Fig. 1C). We found that almost half (47.7%) of the total RBFOX3/NEUN+ neuron population in the midbrain were classed as GABAergic (corresponding to nine clusters: inh-A to inh-E, inh-CCK, inh-PVALB, inh-SST-GAD2^high^, inh-SST-GAD2^low^) and almost a quarter (23.94%) were classed as glutamatergic neurons (corresponding to five clusters: exc-VGLUT2-A to -D, and exc-VGLUT1 and 2) (Fig. 1C and D). Two clusters or 26.28% of nuclei were annotated as mixed clusters or ‘mix-A’ and ‘mix-B’ (Fig. 1C and D). These ‘mixed’ clusters contained nuclei that expressed transcripts for *SLC17A6/*VGLUT2 or *GAD2* (Supplementary Fig. 1B), and importantly, these transcripts were not typically co-expressed by the same nuclei (Supplementary Fig. 1C). These ‘mixed’ clusters may reflect a heterogeneous mixture of various inhibitory and excitatory transcriptional profiles, that could be examined by more detailed subclustering.

Owing to low or neglectable expression of NEUN in DA neurons,^34^ only 398 neurons, or 1.26% of the total NEUN+ population were identified as DA neurons (Fig. 1D), consistent with the relatively low *RBFOX3* mRNA (encoding NEUN) detected (Supplementary Fig. 1D). We found only 0.82% (261) of neuronal nuclei that had a transcriptional profile consistent with serotonergic neurons (Fig. 1C and D). Similar to DA neurons, on the transcriptional level, the neurons annotated as serotonergic, exc-VGLUT2-C, inh-SST-GAD2^high^ and inh-PVALB neurons also had relatively low *RBFOX3* mRNA compared to other neuronal clusters (Supplementary Table 2 and Fig. 1D).

All clusters expressed well-known neuronal markers, such as *SNAP25* and *MAP2* transcripts (Supplementary Fig. 1D). Expression of cell type-specific mRNAs of other CNS cell types, like myelin-associated oligodendrocyte basic protein *MOBP* (oligodendrocytes), aquaporin 4 *AQP4* (astrocytes), purinergic receptor P2Y12 *P2RY12* (microglia), was not detected, confirming that a pure neuronal population was extracted from the tissue (Supplementary Fig. 1D). There was no evidence that clustering results were driven by a nuclear isolation batch or by an individual case (Supplementary Fig. 1E).

To validate cluster annotation, we projected the 18 neuronal clusters to a previously published atlas of the adult human brain.^26^ This publicly available atlas was processed to computationally extract 72 541 midbrain neurons, consisting primarily of neurons annotated as ‘splatter, ‘midbrain-derived inhibitory, and ‘thalamic excitatory’ neurons (Supplementary Fig. 2A). Mapping 18 neuronal clusters onto the midbrain neurons from Siletti *et al*.^26^ resulted in overall high prediction scores (Supplementary Fig. 2B), indicating a high likelihood that 17 out of 18 neuronal clusters identified in the current study are also present in the published brain atlas. Comparing the annotation of superclusters^26^ to the current annotations (Supplementary Fig. 2C) suggested that mix-A, mix-B, and various other clusters corresponded to ‘splatter neurons’ and that the current annotations are more detailed than the supercluster annotation.

Summarizing, snRNAseq of NEUN+ nuclei indicated that the human ventral midbrain is constituted of approximately 18 major neuronal populations, which can be divided into nine inhibitory clusters, five excitatory, two ‘mixed’, and two monoaminergic clusters.

### Diversity in inhibitory midbrain neurons

To determine transcriptional signatures of neuronal midbrain populations, differentially expressed genes (DEGs) were identified for each neuronal cluster compared to all neuronal clusters (Supplementary Table 2). Because we sought transcripts that were robustly marking each cluster, mRNAs were considered differentially enriched markers when the average log2 fold change was > 0.25 and the adjusted *P*-value < 0.05 (Supplementary Table 3). Per cluster, GO terms associated with cluster markers are provided in Supplementary Table 4, confirming the neuronal identity of clusters as indicated by GO terms such as ‘synapse assembly’, ‘axon development’, ‘synaptic vesicle exocytosis’ and ‘neurotransmitter transport’. Among the nine neuronal clusters identified as GABAergic, three clusters (inh-A, inh-B, and inh-D) had similar transcriptional profiles. Nevertheless, they differed in the abundance of certain transcripts such as neuropeptide S receptor 1 (*NPSR1*) for inh-B and hedgehog interacting protein (*HHIP*) for inh-D (Fig. 1E and Supplementary Table 3). Comparing the annotation of a publicly available atlas^26^ to the current clusters suggested inh-A, inh-B, and inh-D corresponded to neurons annotated as ‘midbrain-derived inhibitory’ neurons (Supplementary Fig. 2C).

The GABAergic neuronal cluster inh-PVALB had the most distinct gene expression profile with 1079 enriched cluster markers (avg log2 fold change > 0.25, adjusted *P*-value < 0.05) (Supplementary Table 3). Among the top 50 most significantly enriched transcripts in the inh-PVALB cluster was secretagogin (*SCGN*), encoding a calcium-binding protein, and *GRM1*, which codes for metabotropic glutamate receptor 1 (Fig. 1E and Supplementary Table 3). In inh-*SST-GAD2*^high^, the neuronal cluster with the most abundant somatostatin (*SST*) expression, we observed enriched expression of prospero homeobox 1 (*PROX1*), neuron derived neurotrophic factor (*NDNF*) and cadherin 20 (*CDH20*) in the top 50 most significantly enriched transcripts (Fig. 1E and Supplementary Table 3).

Inh-CCK was characterized by significantly enriched expression of *DLX6-AS1,* reelin *(RELN),* C-X-C motif chemokine ligand 14 (*CXCL14*), erb-b2 receptor tyrosine kinase 4 (*ERBB4*), adenosine deaminase RNA specific B2 (*ADARB2*), and distal-less homeobox 1 (*DLX1*) within the top 50 of most significantly enriched genes (Fig. 1E and F and Supplementary Table 3). Lysosomal associated membrane protein family member 5 (*LAMP5*), a marker for interneurons derived from the caudal ganglionic eminence during development, did not rank among the top 50 most significant cluster marker genes but was exclusively expressed in the inh-CCK neuronal cluster (Fig. 1E and Supplementary Table 3).

The transcriptomic data was orthogonally validated by immunohistochemical stainings. *DLX1*, a marker gene for the inh-CCK cluster (Fig. 1F), positively stains nuclei with a neuronal morphology (brown staining), but not astrocytes (GFAP, black staining), in the periaqueductal grey, the nucleus of the third cranial nerve and the reticular formation of the midbrain (Fig. 1G and H).

ETS variant transcription factor 5 (*ETV5*) is a significantly enriched marker gene for the large inh-B cluster and is also expressed in the DA and serotonergic clusters (Supplementary Fig. 2D). Consistent with our transcriptomic results, many non-astrocytic nuclei (brown staining), were labelled for ETV5 throughout different midbrain regions including pigmented cells of substantia nigra (Supplementary Fig. 2E and F). The DLX1 and ETV5 signals were found in different anatomical areas of the human midbrain, validating our identification of these markers for neuronal populations as predicted by our snRNAseq data.

### Diversity in excitatory midbrain neurons

Characterizing VGLUT2-expressing neurons in the midbrain is crucial, as glutamatergic VTA neurons project extensively to the prefrontal cortex and limbic structures, influencing critical neuronal circuits involved in learning, mood, motivation, and addiction behaviour.^35^ In NEUN+ midbrain neurons, we identified five glutamatergic neuronal populations (Fig. 1B and C). Exc-VGLUT2-A, the largest glutamatergic neuron population (11.18% of total and 46.73% of glutamatergic clusters) had significantly enriched expression of the transcription factor basonuclin zinc finger protein 2 (*BNC2*) (Fig. 1E). A subset of nuclei in exc-VGLUT2-B, C, and D clusters co-expressed *PVALB* and *SLC17A6,* encoding PV and VGLUT2 respectively (Supplementary Fig. 2G). However, clustering did not separate the *SLC17A6* + *PVALB* + nuclei, indicating their close transcriptional relationship to *SLC17A6* + *PVALB*- nuclei.

A small population of glutamatergic neurons co-expressed both *SLC17A6* and *SLC17A7*, encoding VGLUT1 and -2. This dual expression led to their annotation as exc-VGLUT1 and 2 neurons (Supplementary Fig. 2G). Transcripts among the top 50 most significantly enriched in these exc-VGLUT1 and 2 neurons included neuregulin 1 (*NRG1*), potassium voltage-gated channel Q5 (*KCNQ5*), dipeptidyl peptidase like 6 (*DPP6*), glypican 5 (*GPC5*) (Supplementary Table 3). Cytochrome P450 family 26 subfamily B member 1 (*CYP26B1*), an enzyme involved in regulating retinoic acid levels in the brain,^36^ did not rank among the top 50 most significant markers, but was exclusively expressed in the exc-VGLUT1 and 2 cluster (Supplementary Fig. 2H). The presence of exc-VGLUT1 and 2 in the midbrain was corroborated at the protein level by immunohistochemical stainings. CYP26B1 signal (light brown) was detected in the periaqueductal grey and midline region, distinct from GFAP-positive cells (black) in control midbrain sections (Supplementary Fig. 2I) suggesting that neuron-like cells express this protein.

### A subtype of *RBFOX3*^low^ dopaminergic neurons expresses aldehyde dehydrogenase 1 A2

Although previous studies report limited NEUN expression in DA neurons,^34^ our analysis identified a small proportion of DA neurons using RBFOX3/NEUN+ sorting. Defining the diversity within DA neurons remains relevant since NEUN is a widely used immunohistochemical marker. Here, we defined DA neurons as nuclei that are co-expressing both tyrosine hydroxylase *TH* and the dopamine transporter *SLC6A3*/DAT. This constituted a subgroup of 123 DA neurons within the larger cluster annotated as ‘dopaminergic’, indicating that the DA cluster contained additional transcriptional heterogeneity. Focusing on the 123 ‘classical’ DA neurons, most of these neurons also had abundant expression of vesicular monoamine transporter 2 (*SLC18A2*) and dopamine receptor 2 (*DRD2*) but low average *RBFOX3* expression (*RBFOX3*/NEUN^low^) compared to non-DA neurons (Supplementary Fig. 3A).

Within the total 123 DA transcriptional profiles, two subpopulations of DA neurons could be identified depending on their expression of aldehyde dehydrogenase 1 family member A2 mRNA whose enzyme product makes retinoic acid (RA): *RBFOX3*^low^*ALDH1A2*^neg^ and *RBFOX3*^low^*ALDH1A1*^pos^ (Supplementary Fig. 3B and Supplementary Table 5). These two populations of dopamine neurons have similar expression levels of *SLC18A2*, *TH*, *SLC6A3* and *DRD2* mRNAs (Supplementary Fig. 3C). *ALDH1A1*-expressing DA neurons are reported to have an alternative dopamine metabolism, different electrophysiological properties, and are spatially restricted to the ventral SNpc.^37^ To compare the two distinct DA neuronal profiles (*RBFOX3*^low^*ALDH1A2*^neg^ and *RBFOX3*^low^*ALDH1A1*^pos^), gene expression analysis between these subpopulations of DA neurons was performed (Supplementary Table 5). Thyrotropin-releasing hormone receptor (*TRHR*) and SAMD family member 3 (*SAMD3*) were transcriptional markers enriched in *RBFOX3*^low^*ALDH1A1*^neg^ DA neurons (Supplementary Fig. 3D and Supplementary Table 6). Regulator of G protein signalling 6 (*RGS6*) and netrin receptor (*DCC*) were identified as transcriptional markers for *RBFOX3*^low^*ALDH1A1*^pos^ DA neurons (Supplementary Fig. 3E and Supplementary Table 6).

### Heterogenous neuronal profiles cluster in the ‘mixed’ clusters

**Figure 2:**
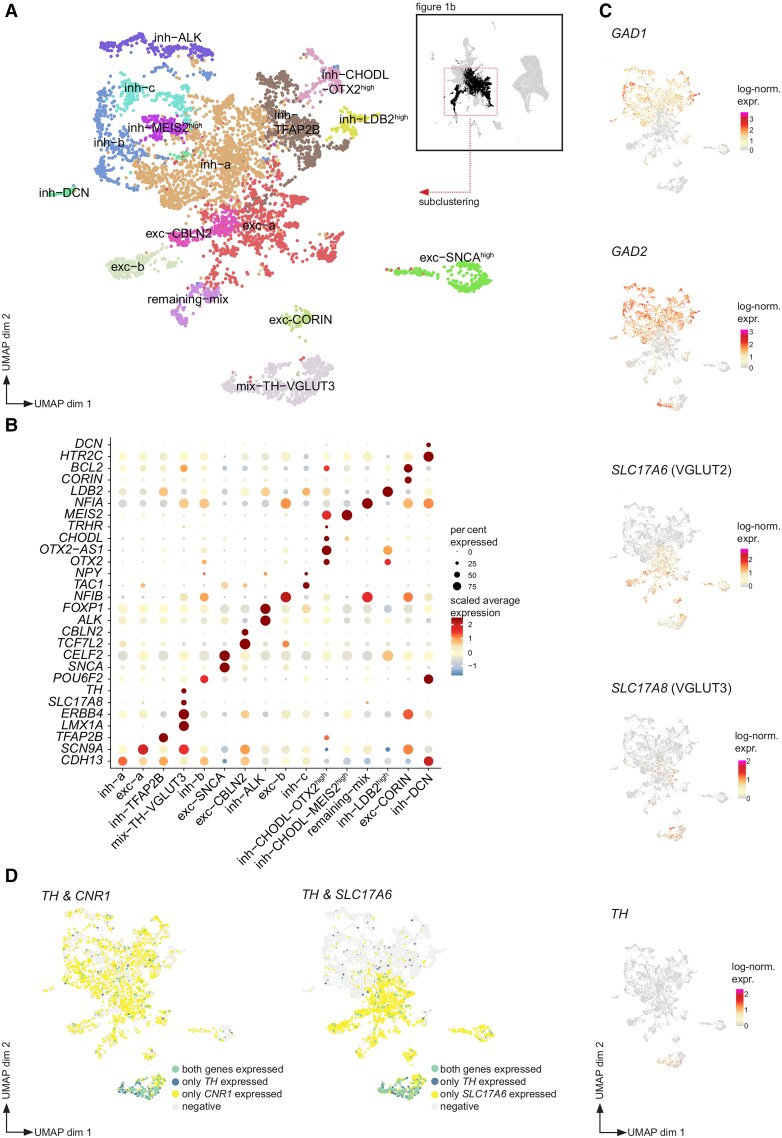
**Subclustering of mixed clusters identifies 16 additional neuronal subpopulations**. (**A**) Unsupervised subclustering of 6654 nuclei corresponding to mix-A and mix-B. Each dot indicates a nucleus, and colours indicate subclusters (Supplementary Tables 7 and 8). (**B**) Dot plot depicting marker genes per subcluster. Size of the circles indicates the percentage of nuclei expressing the gene, colour indicates the scaled average gene expression (Supplementary Table 8). (**C**) Uniform Manifold Approximation and Projection (UMAP) depicting the expression of neurotransmitter-related genes (*GAD1*, *GAD2*, *SLC17A6*, *SLC17A8*, *TH*). Colour indicates log-normalized gene expression. (**D**) UMAPs depicting the co-expression of *TH* and *CNR1* (*left*) and *TH* and *SLC17A6* (*right*). expr. = expression; log-norm = log-normalized.

In nuclei annotated as ‘mixed’, co-expression of *GAD2* and *SLC17A6* was detected in only ∼2% of the NEUN+ neuronal midbrain population. The presence of different neurotransmitter-related genes in the same nuclear cluster and a lack of co-expression in many other nuclei within the ‘mixed’ clusters suggested that the clusters annotated as mix-A and mix-B may consist of distinct inhibitory and excitatory neuronal subpopulations. To investigate if and how inhibitory neurons were distinct from excitatory neurons in the mixed clusters, we performed a more fine-grained subclustering of 6654 nuclei corresponding to two of our original clusters (mix-A and mix-B; Fig. 2A, inset). After QC (Supplementary Fig. 4A and B), the subclustering revealed 16 additional neuronal subpopulations (Fig. 2A and Supplementary Table 7). These 16 subpopulations had discrete identities, as indicated by the number (≥170) of differentially expressed genes detected per subcluster (Fig. 2A and B and Supplementary Table 8).

Inh-*CHODL-OTX*^high^, a subpopulation that highly expressed chondrolectin (*CHODL),* thyrotropin-releasing hormone receptor (*TRHR*), orthodenticle homeobox 2 antisense RNA 1 (*OTX2-AS1*), and orthodenticle homeobox 2 (*OTX2*) (Fig. 2B), was enriched in GO terms such as ‘modulation of chemical synaptic transmission’ and ‘axonogenesis’ (Supplementary Table 9). OTX2 is a transcription factor specifically expressed in the ventral parts of the rodent VTA.^38^

Subpopulation inh-C had enriched expression of tachykinin precursor 1 (*TAC1*) (Fig. 2B). Transcripts for *TAC1* encode neurokinin A and substance P.^39^ GO terms associated with cluster markers of the inh-C subpopulation included ‘neuropeptide signaling pathway' (Supplementary Table 9). In addition, the inh-C subpopulation had enriched, albeit not exclusive, expression of neuropeptide Y (*NPY*) (Fig. 2B).

We observed that a subset of TH + SLC6A3- nuclei initially assigned to mix-B, now grouped in a new cluster annotated as ‘mix-TH-VGLUT3’. The nuclei in mix-TH-VGLUT3 expressed *LMX1A*, a marker of mature DA neurons, vesicular monoamine transporter 2 (*SLC18A2*), dopamine receptor 2 (*DRD2*), and Nuclear Receptor 4A2 (*NR4A2*) (Supplementary Table 8), which is a recently identified transcription factor specific to mammalian midbrain DA neurons.^40^ However, the mix-TH-VGLUT3 cluster did not express the dopamine transporter gene (*SLC6A3/*DAT1) (Supplementary Fig. 4C). In mix-TH-VGLUT3, the expression of *GAD2,* vesicular glutamate transporter 2 *(SLC17A6/*VGLUT2*),* and vesicular glutamate transporter 3 (*SLC17A8/*VGLUT3) mRNA transcripts were specific to certain compartments of the cluster in the two-dimensional UMAP (Fig. 2C), indicating additional heterogeneity in *TH*-expressing neurons. Approximately 33% (177 nuclei) of nuclei within the mix-TH-VGLUT3 cluster co-expressed transcripts for *TH* and the cannabinoid CB1 receptor (*CNR1*) and co-expressed *TH* and *SLC17A6* (Fig. 2D), thereby suggesting this may be a subset of DA neurons that is characterized by the ability to co-release dopamine and glutamate.^41^ This subset is described as a distinct DA population that is mainly located in the midsection of the rodent VTA and triple positive for *CNR1*, *TH* and *SLC17A6* using in situ hybridization.^41^

### Subtle shifts in proportions of midbrain neuronal subpopulations in schizophrenia

To investigate the schizophrenia-associated transcriptional changes in the midbrain neurons, we investigated the neuronal gene expression profiles in schizophrenia cases on multiple levels: the relative abundance of neuronal populations (Fig. 1B), the relative abundance of neuronal subpopulations (Fig. 2A), the transcriptional changes per neuronal (sub)population in schizophrenia, and the expression of schizophrenia-related GWAS-identified genes in our dataset.

**Figure 3:**
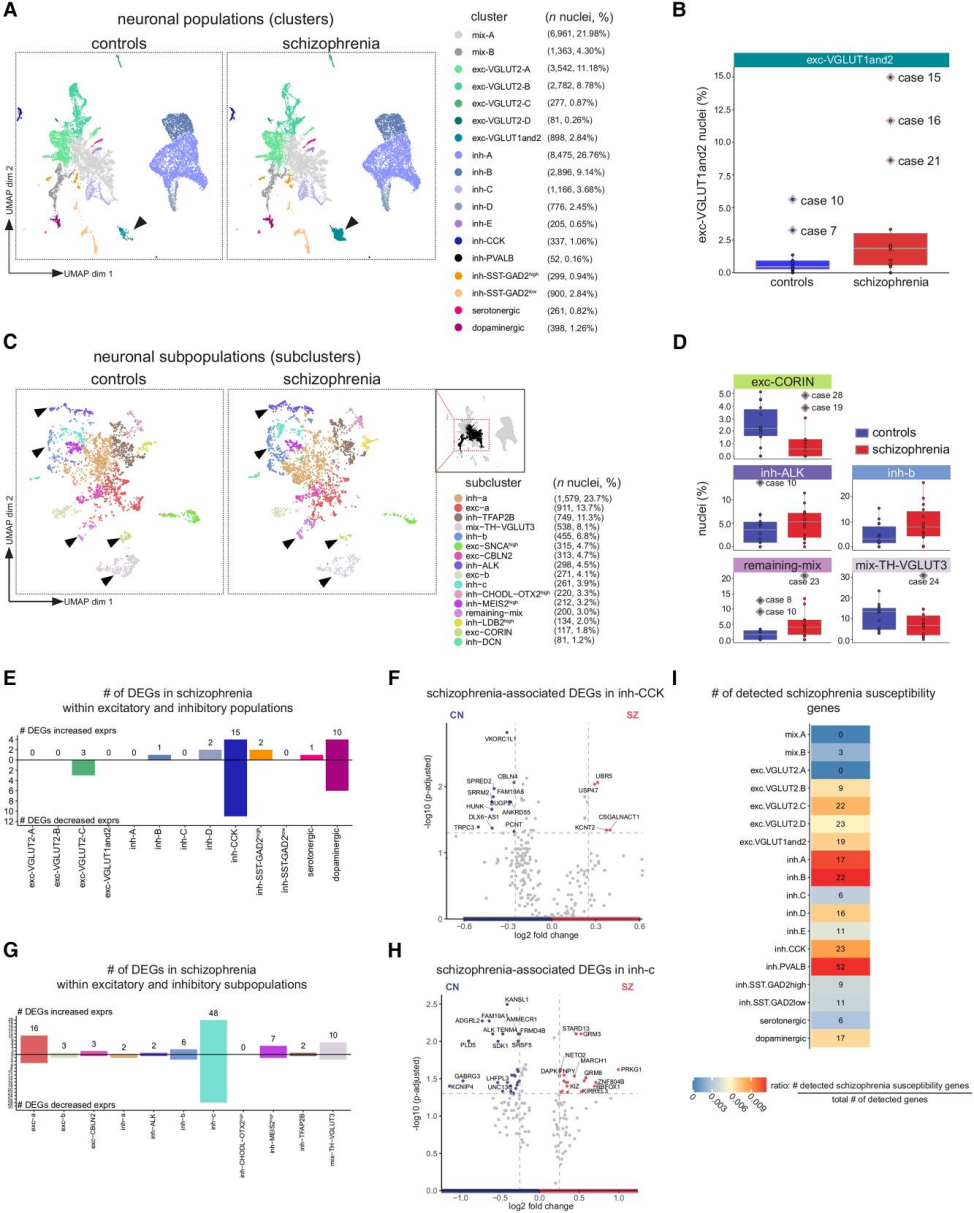
**Schizophrenia-associated shifts in proportions of midbrain neuronal populations and subpopulations**. (**A**) Uniform Manifold Approximation and Projection (UMAP) of the clusters split by diagnosis. The colour legend indicates the number of nuclei obtained per population and the percentage of the total NEUN+ population enclosed in brackets. (**B**) Box plot depicting the percentage of exc-VGLUT1 and 2 nuclei detected in midbrain samples of schizophrenia and control cases (*n* = 14 per group; Supplementary Table 10). Outlier cases are labelled with grey diamond shapes, each dot represents a case, and horizontal lines indicate the median. Outliers were defined as proportions outside of the third quartile plus 1.5 times the interquartile range. (**C**) UMAP of the subclusters split by diagnosis. The colour legend indicates the number of nuclei obtained per population and the percentage of the total NEUN+ population enclosed in brackets. (**D**) Box plots depicting the percentage of nuclei detected per case in subclusters that had significantly altered proportions in schizophrenia; Supplementary Tables 10 and 12. Outlier cases are labelled with grey diamond shapes and were defined as data points outside the third quartile + 1.5 × interquartile range. (**E**) Total count of differentially expressed genes (DEGs) between schizophrenia and controls within a neuronal population. Numbers above the bar indicate the total count of increased plus decreased gene expression changes in schizophrenia. (**F**) Volcano plot depicting differentially expressed genes (absolute log_2_ fold-change > 0.25, *P*-adjusted < 0.05) between schizophrenia and controls within the neuronal population ‘inh-CCK’. The corresponding information on DEGs is available in Supplementary Table 13. (**G**) Total count of differentially expressed genes between schizophrenia and controls within a neuronal subpopulation. Numbers above the bar indicate the total count of increased plus decreased gene expression changes in schizophrenia. (**H**) Volcano plot depicting DEGs (absolute log_2_ fold-change > 0.25, *P*-adjusted < 0.05) between schizophrenia and controls within the neuronal subpopulation ‘inh-C’. The number of gene labels was reduced to avoid overlapping text; the full information on DEGs is available in Supplementary Table 14. (**I**) Heat map with the number indicating the detected schizophrenia susceptibility genes per neuronal population (Supplementary Table 16). The colour indicates the normalized ratio of detected susceptibility genes over the total number of expressed genes per neuronal population. High-confidence schizophrenia susceptibility genes derived from Wang *et al*.^27^

We determined if the relative abundance of NEUN+ midbrain neuron populations differed in schizophrenia when compared to controls (Supplementary Table 10). When using a Bayesian model for compositional analysis, which jointly models the contribution of schizophrenia and control cells to all clusters,^31^ no differences in neuronal cluster composition between schizophrenia and control cases were detected (Supplementary Table 11). However, when using a generalized linear model that individually models each cluster for the contribution of schizophrenia and control cells, subtle, putative differences could be identified (Supplementary Table 12). Among the 18 clusters, the most substantial increase in proportion was detected for neurons classified into the VGLUT1 and 2 excitatory cluster in patients with schizophrenia compared to controls (*P*-value = 0.011, *n* = 14 cases per group) (Fig. 3A and B, Supplementary Fig. 5A and Supplementary Table 12). After controlling the false discovery rate with a predetermined FDR threshold of 0.2, we found that this increase in the proportion of VGLUT1 and 2 excitatory neurons in patients with schizophrenia was at the threshold level of statistical significance (Supplementary Table 12). In absolute numbers, the total number of exc-VGLUT1 and 2 neurons was approximately 5 times higher in schizophrenia cases (*n* = 752) than in controls (*n* = 146). The relatively small average percentage change overall (1.4%) in VGLUT1 and 2 excitatory neurons in schizophrenia cases could not be explained by case-related variables such as sex, nuclear isolation batch, brain pH, age, or estimated medication usage (Supplementary Fig. 6A).

We adhered to the identical procedure to test for a difference in the respective contribution of schizophrenia and controls to neuronal subpopulations (Supplementary Fig. 5B and Supplementary Tables 10–12). Neuronal subpopulations are defined as the populations identified by subclustering of the ‘mixed’ clusters (Fig. 2A). Using compositional analysis through scCODA, no differences in neuronal subcluster composition between schizophrenia or control cases could be identified (Supplementary Table 11). When using a generalized linear model to investigate the contribution of schizophrenia and control cells to each individual cluster, and after applying the Benjamini-Hochberg correction, five subpopulations had significantly altered abundance among individuals with schizophrenia (Fig. 3C and D). Neurons classified as ‘inh-ALK’ (*P*-value = 0.017), ‘inh-B’ (*P*-value = 0.023), and ‘remaining-mix’ (*P*-value = 0.035) were more abundant in schizophrenia, while those classified as ‘exc-CORIN’ (*P*-value = 0.017) and ‘mix-TH-VGLUT3’ (*P*-value = 0.05) were less abundant in schizophrenia compared to controls (*n* = 14 cases per group, FDR < 0.2) (Fig. 3D and Supplementary Table 12). Each neuronal subpopulation was relatively small in numbers, constituting on average 4.8% of the total ‘mixed’ population. The significant changes in proportions of exc-CORIN, inh-ALK, remaining-mix, and mix-TH-VGLUT3 were not associated with case-related variables such as brain pH, age, estimated medication usage, sex, or nuclear isolation batch (Supplementary Fig. 6B). Changes in the proportions of neurons classed to the inh-B subpopulation negatively correlated with age (Pearson’s correlation = −0.45, *P*-value = 0.017), suggesting the relative abundance of neurons classified as inh-B decreased with older age (Supplementary Fig. 6C). However, inh-B proportions were not associated with other case-related variables sex, nuclear isolation batch, estimated medication usage, or brain pH (Supplementary Fig. 6C).

### Schizophrenia-associated transcriptional changes in inhibitory neuronal populations

To investigate gene expression differences between control and schizophrenia cases, gene expression profiles within neuronal populations were compared. This approach could identify gene expression changes occurring only within a (sub)population, which could remain undetected in bulk RNA sequencing analysis. Within each neuronal population, we assessed transcriptomic differences between schizophrenia and controls. We excluded exc-VGLUT-D, inh-E, and inh-PVALB due to insufficient case and nuclei counts (less than seven cases with more than 10 nuclei per case). Mix-A and mix-B were omitted as individual subclusters were investigated in detail in the next analysis. Given the number of comparisons, we applied strict FDR correction (FDR < 0.05), and only 33 differentially expressed genes (DEGs) were identified, 20 of which were within an inhibitory neuronal population (Fig. 3E and Supplementary Table 13).

The highest number of gene expression changes, in total 15, were observed in inh-CCK neurons, including decreased transient receptor potential cation channel subfamily C member 3 (*TRPC3*) expression and increased chondroitin sulfate *N*-acetylgalactosaminyltransferase 1 (*CSGALNACT1*) expression (Fig. 3F and Supplementary Table 13). The most pronounced gene expression changes (largest log_2_-fold changes) were detected in the DA population (Supplementary Fig. 7A and Supplementary Table 13). RBFOX3/NEUN^low^ DA neurons had increased expression of ectonucleoside triphosphate diphosphohydrolase 1 antisense RNA 1 (*ENTPD1-AS1*) and dopamine transporter 1 (*SLC6A3*/DAT1) in schizophrenia cases (Supplementary Fig. 7A and Supplementary Table 13). Furthermore, the DA population in schizophrenia had decreased expression of genes associated with the GO term ‘GTPase activity’ (Supplementary Fig. 7B), such as Ras-related protein Rab-27B (*RAB27B*), and ADP ribosylation factor (*ARL17B*). In short, we identified 33 differentially expressed genes in schizophrenia, and almost two-thirds of DEGs were detected within GABAergic midbrain neurons, but the largest gene expression increase (in log fold change) was detected in the DA cluster.

Next, we investigated the transcriptional differences that could occur within the subpopulations identified by subclustering of ‘mixed’ neuronal populations (Fig. 2A). The subpopulations exc-CORIN, exc-SNCA^high^, inh-DCN, inh-LDB2^high^, and remaining-mix were excluded, due to insufficient cases providing more than 10 nuclei per case. Within the analysed subpopulations (exc-a, exc-b, exc-CBLN2, inh-A, inh-ALK, inh-B, inh-C, inh-CHODL-OTX2^high^, inh-MEIS2^high^, inh-TFAP2B, mix-TH-VGLUT3) a total of 99 DEGs were identified, of which 67 were within an inhibitory neuronal subpopulation (Fig. 3G and Supplementary Table 14). Neurons classified into the inh-C cluster had the highest number of gene expression alterations, in total 48 (Fig. 3G and H). Among all neuronal subpopulations, the largest gene expression increase was observed for protein kinase CGMP-dependent 1 (*PRKG1*) (Fig. 3H and Supplementary Table 14), which is associated with the GO term ‘calcium channel regulator activity’ (Supplementary Fig. 7D) in inh-C neurons. Inh-C neurons also had increased expression of metabotropic glutamate receptors *GRM3* and *GRM8* in schizophrenia compared to controls (Fig. 3H and Supplementary Table 14), associated with the GO term ‘glutamate receptor activity’ (Supplementary Fig. 7D), and decreased expression of potassium voltage-gated channel interacting protein 4 (*KCNIP4*) and GABA-receptor subunit gamma 3 (*GABRG3*) (Fig. 3H and Supplementary Table 14). Interesting to note were the differentially expressed genes in mix-TH-VGLUT3 neurons in schizophrenia (in total 10; Fig. 3G), including increased expression of glypican 6 (*GPC6*) in schizophrenia compared to controls (Supplementary Fig. 7C and Supplementary Table 14). Two differentially expressed genes associated with schizophrenia, *RGS6* and *PTPRK*, are detected within the exc-a subcluster and are recognized as high-confidence susceptibility genes for schizophrenia (Supplementary Fig. 7E and Supplementary Tables 14 and 15).

Concluding, transcriptional changes between schizophrenia and control cases are most pronounced in inhibitory neuronal (sub)populations. Possibly, new genes (*RGS6* and *PTPRK*) have been identified in an excitatory neuronal subpopulation that might be important in the pathophysiology of schizophrenia.

### The highest number of schizophrenia susceptibility genes is detected in inhibitory neuron populations

To connect GWAS results for schizophrenia to specific neuronal midbrain populations, we mapped the expression of schizophrenia GWAS genes onto the single-nucleus clusters (Fig. 3I). We used a curated GWAS gene set denoted as ‘high-confidence’, which was supported by more than two evidence sources in PsychEncode Consortium analysis^27^ (Supplementary Table 15). Genes were defined as detected in a population if the *z*-score of the average expression in the population was greater than 1.5. Inh-PVALB, inh-B and inh-A had the highest ratios, which are calculated by detected schizophrenia genes divided by the total number of detected genes per cluster (Fig. 3I). The highest number of schizophrenia susceptibility genes (52 genes) were detected in the population of inh-PVALB neurons compared to other midbrain neurons. Taking both the large number of genes detected in inh-PVALB neurons and the multiple populations that were tested into account, the overlap between the GWAS-identified gene set (213 genes) and expressed genes in inh-PVALB neurons (4619 genes) was not significant (Fisher’s exact test, *P* = 0.018, *P*-adj = 0.32). The schizophrenia susceptibility genes detected in inh-PVALB neurons in the midbrain included genes such as *DGKZ*, *INPP4B* and *TKT* (Supplementary Table 16). The schizophrenia susceptibility genes detected in inh-PVALB were associated with the KEGG Pathway ‘Phosphatidylinositol signalling pathway’ and GO terms ‘lipid modification’ and ‘glycerolipid metabolic process’ (Supplementary Table 16). To summarize, inh-PVALB, inh-B and inh-A had the highest ratio of detected schizophrenia susceptibility genes in neurons analysed from these 28 cases.

## Discussion

Midbrain samples from 14 schizophrenia and 14 control cases obtained from the Stanley Medical Research Institute (SMRI) Array Collection were analysed by sn RNAseq of fluorescence-activated nucleus sorted NEUN+ nuclei. Selected transcriptional markers were confirmed at the protein level with immunohistochemical stainings, validating the existence of neuronal populations predicted by snRNAseq in the midbrain. We studied the ventral midbrain because this is the region where altered functioning of GABAergic, glutamatergic and DA circuits in schizophrenia is likely to occur. We discovered subtly altered relative abundance and transcriptional differences within neuronal populations (clusters) and subpopulations (subclusters) of glutamatergic, GABAergic and DA neurons in schizophrenia by analysing 16 616 nuclei from schizophrenia and 15 053 nuclei from control cases. The relative abundance of neuronal subpopulations was changed in the ventral midbrain of patients with schizophrenia, indicated by subtle proportional shifts in both excitatory and inhibitory neurons. We also detected differences in the transcript levels within neurons between patients and controls, with a preponderance of deviations within the GABAergic neurons, as most transcriptional changes (20 out of 33 for populations, 67 out of 99 for subpopulations) were identified within these inhibitory neurons. Schizophrenia-associated differentially expressed genes in the midbrain and cortex are not overlapping (data not shown), indicating brain region-specific alterations in schizophrenia, as previously described.^42^ This highlights the need for individual analysis of brain regions in schizophrenia research.

The proportion of neurons annotated as excitatory VGLUT1 and 2 neurons was potentially increased in schizophrenia and those annotated as excitatory CORIN+ neurons were reduced compared to controls. This may suggest that the balance of excitatory output of the midbrain is altered in schizophrenia, favoring the information carried by VGLUT1 and 2+ neurons over that carried by CORIN+ neurons. Possibly, these shifts could indicate that in patients with schizophrenia, changes occur in the type of midbrain glutamate input onto other midbrain neurons. In support of this, we detected altered expression of metabotropic glutamate receptors (*GRM3, GRM8*) on inhibitory neurons in subpopulation inh-C and deviant expression of synapse differentiation induced gene 1 (*SYNDIG1*), which encodes an activity-dependent AMPA-receptor auxiliary subunit,^43^ in neurons classified as inh-B. These findings suggest that changes in midbrain glutamate neurons may lead to synaptic changes within the midbrain.

At the transcription level, gene expression changes in schizophrenia were mostly detected within the low abundant GABAergic (sub)populations, including inh-CCK and inh-C. Additionally, we found that inhibitory *PVALB*-expressing neurons expressed many schizophrenia risk genes. Multiple differentially expressed genes (*KCNIP4*, *GRM3*, *PRKG1*, *NPY* for inh-C, and *TRPC3* for inh-CCK) were related to glutamate reception and calcium channel activity, indicating these processes are altered within inhibitory midbrain neurons in schizophrenia. Such alterations may lead to decreased or otherwise deviant excitability of these inhibitory neurons which subsequently may influence DA neuron activity either directly or indirectly.^14^ Dopamine hyperactivity is known as a final common pathway to give rise to psychotic vulnerability, the hallmark of schizophrenia.^44-47^

Contrary to the hypothesis of decreased inhibitory influence in the midbrain, we found that the proportions of two subpopulations of inhibitory neurons (inh-ALK and inh-B) were potentially increased in schizophrenia, which may indicate a shift in the type of inhibitory control within the ventral midbrain, rather than a loss of inhibitory neurons.

The proportion of neurons co-expressing transcripts for *TH* and vesicular glutamate transporters was reduced with marginal significance in schizophrenia compared to controls. Since these DA neurons co-expressed glutamate transporters, they may correspond to VTA dopamine neurons that project to limbic regions and to the prefrontal cortex.^48,49^ This aligns with the loss of dopamine innervation that has been reported in the prefrontal and temporal cortex in patients with schizophrenia.^50,51^ In support of reduced dopamine synthesis capability in the schizophrenia midbrain, less *TH* mRNA and TH protein were measured in the midbrain of patients with schizophrenia by us and others.^15,52,53^ However, results of no change or an increase in *TH* in the schizophrenia midbrain have also been reported.^45,54,55^ We emphasize that we examined NEUN+ dopamine neurons, representing only a subset of dopamine neurons, including those expressing *ALDH1A1* and others that may co-synthesize glutamate. This dopamine-glutamate co-transmission has been linked to salience attribution in response to environmental cues,^48,56^ which is known to be aberrant in patients with psychosis.^8,57^ While the need for additional validation remains evident, particularly for the *TH*-expressing populations described here, our study suggests the loss of a potentially vulnerable subpopulation of DA neurons, which may also co-transmit glutamate in patients with schizophrenia.

‘Mixed’ neuronal populations uniquely comprised nuclei expressing GABA- and glutamate-related genes and did not separate on the two-dimensional UMAP plot. A similar neuronal population was reported in an adult human brain atlas, referring to these neurons as ‘splatter neurons’.^26^ Computational projection of ‘mixed’ clusters onto the adult human brain atlas confidently assigned ‘mixed’ clusters as ‘splatter neurons’. Splatter/mixed neurons were transcriptionally more heterogeneous than any other neuronal population from the same dissection, were reproducibly identified, and were not due to quality metrics like total UMI counts. The midbrain, thalamus, and hindbrain regions had the highest relative abundance of ‘splatter neurons’, while the lowest abundance was observed in the telencephalon.^26^ More schizophrenia-associated transcriptional changes were identified in our subpopulations of splatter/mixed neurons than were identified in the main populations. This underscores the importance of investigating the phenotypes and functions of ‘splatter neurons’ in future schizophrenia research.

Two additional studies have applied snRNAseq to investigate the human midbrain, focusing on total cellular diversity resulting in a relatively limited number of neurons in the data (*n* = 5332 neuronal nuclei in Smajic *et al.*^20^ and *n* = 195 in Agarwal *et al.*^58^), whereas we have examined over 30 000 NEUN+ nuclei demonstrating that selecting neurons based on marker proteins is advantageous. Indeed, a recent snRNAseq study used sorting with a selective dopamine neuron marker NR4A2 (rather than NEUN) to examine over 300 000 midbrain nuclei (22 000 dopamine neurons) and identified a subpopulation of dysfunctional ventral tier DA neurons associated with Parkinson’s disease.^40^ Complementary, we analysed high numbers of non-DA neurons in the human ventral midbrain. This allowed us to investigate less common GABAergic cell populations, making a unique contribution to schizophrenia research. Other studies that used a similar technique focused on other brain areas in schizophrenia. For example, Batiuk *et al.*^59^ focused on the dorsolateral prefrontal cortex and found specific upper-layer GABAergic interneurons and lower-layer pyramidal neurons that were transcriptionally affected in schizophrenia. This affirms that schizophrenia-associated changes impact not just one type of neuron, but various neuronal subtypes across distinct brain regions.

Several weaknesses of the current study might limit our identification of schizophrenia-associated transcriptional changes. Foremost, the NEUN+ sorting procedure did not completely capture certain neuronal midbrain populations, such as the serotonergic neurons, PVALB-expressing neurons, and all varieties of DA neurons. This may have limited our ability to detect a previously published^15^ reduction of *GAD1* and *PVALB* transcript levels in schizophrenia samples in this population. Furthermore, the clinical heterogeneity among the donors and the disease state at the time of tissue collection can vary in this schizophrenia cohort. We appreciate that the investigation of a heterogeneous and multifactorial disease such as schizophrenia will benefit from an even higher number of cases, a greater total number of nuclei per case, and more sequencing depth per nucleus. Additionally, we did not take the inflammatory status of the tissue into account in this study.^60^

Differences in subcluster contribution between schizophrenia and control cases hint toward changes in small subclusters in our dataset. Although schizophrenia is a devastating disease that profoundly affects patients’ behaviour, it is not directly life-threatening. Therefore, the subtle cellular changes observed may align with the underlying biology of the disease.

Gradient centrifugation during nuclei isolation separates cytosolic mRNA from mRNA that is captured in the nucleus. Previous studies have shown that nucleic and cytosolic mRNA transcripts in neurons differ quite substantially.^61,62^ Possibly, greater gene expression differences between schizophrenia and control cases could have been identified in the cytosolic mRNA fraction of analysed neurons. As with all novel findings, the biological relevance and transcriptomic changes we observed between schizophrenia and control cases in small subclusters, need to be verified in future studies.

In summary, analysing high numbers of NEUN+ midbrain neurons, we observed that schizophrenia subtly affects the composition of various neuronal types (glutamatergic, GABAergic, and TH-VGLUT3-expressing neurons) and is mostly associated with gene expression changes within specific GABAergic subpopulations. These findings offer novel insights for further investigations, ultimately enhancing our understanding of the molecular basis of schizophrenia.

## Supplementary Material

awae321_Supplementary_Data

## Data Availability

The authors confirm that the data supporting the findings of this study are available within its Supplementary material and Figures. Raw data are available through the SMRI website, www.stanleyresearch.org, or directly at http://sncid.stanleyresearch.org/.
