# Supplementary material for: Schizophrenia-associated changes in neuronal subpopulations in the human midbrain
Source: Brain. 2024 Oct 14;148(4):1374–88. doi: 10.1093/brain/awae321 (PMC11969452; doi:10.1093/brain/awae321)
Supplement: awae321_Supplementary_Data [file awae321_supplementary_data.zip › brain-2023-02291-File005.pdf]

Supplementary Figure 1

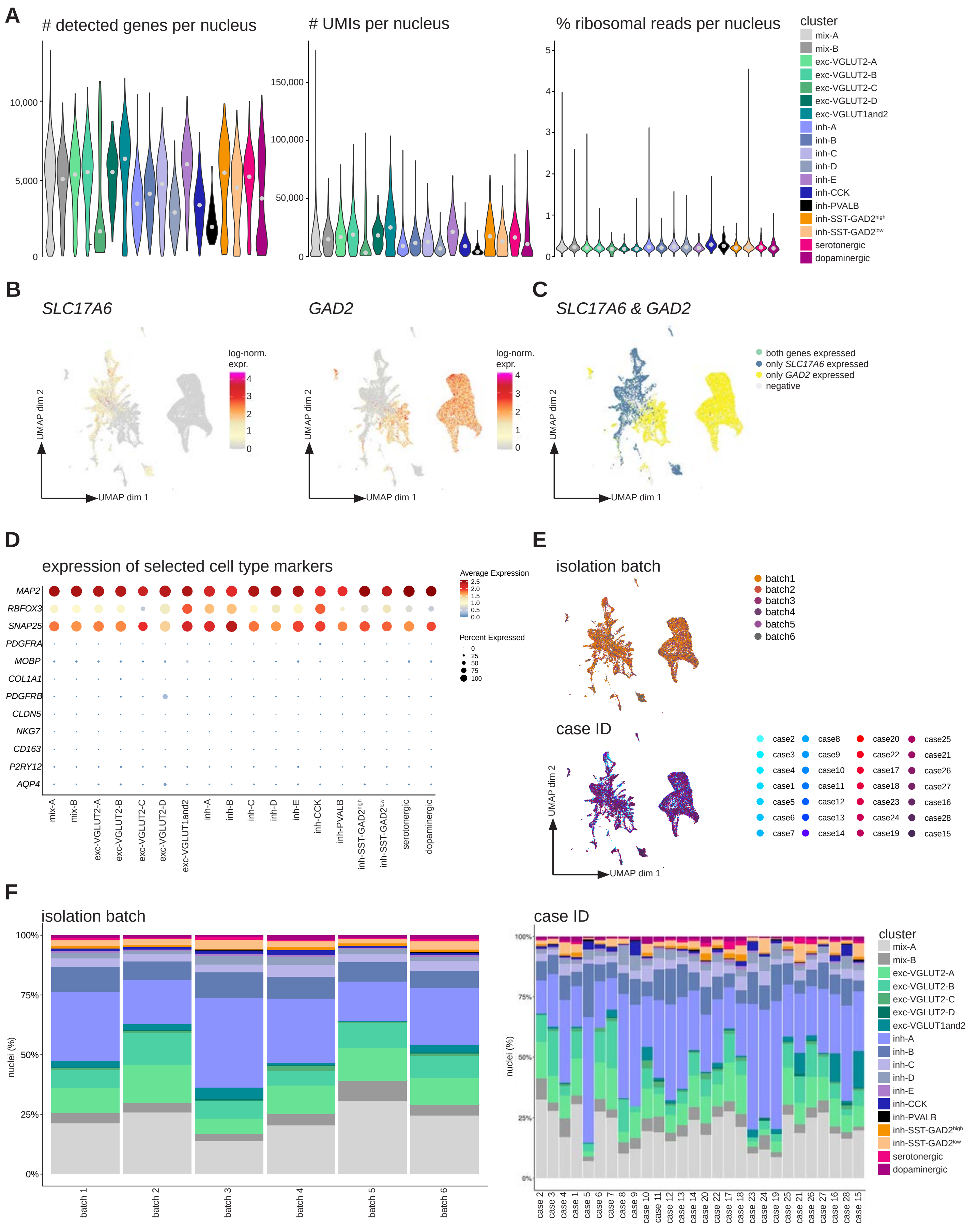

Supplementary Figure 2

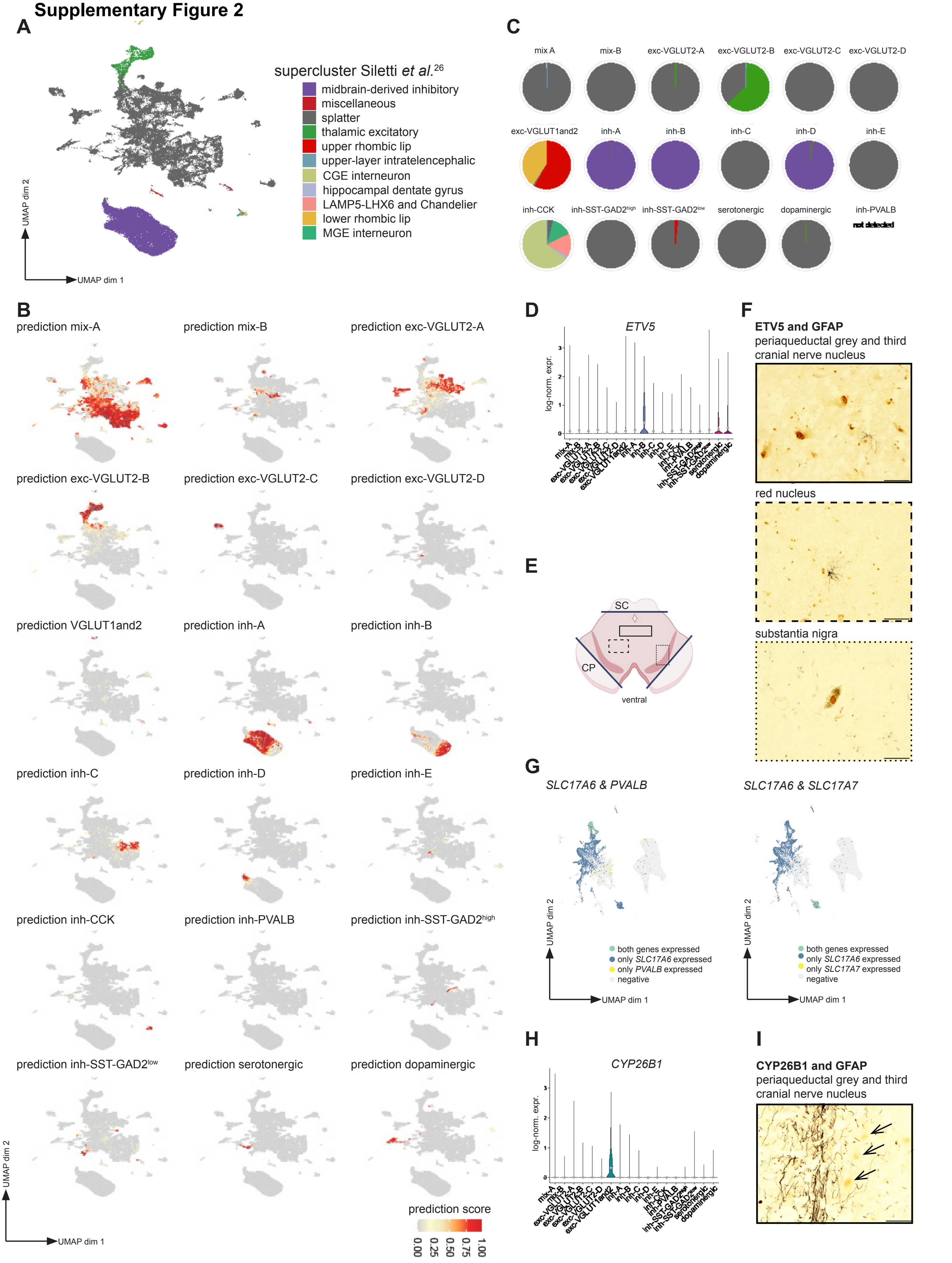

Supplementary Figure 3

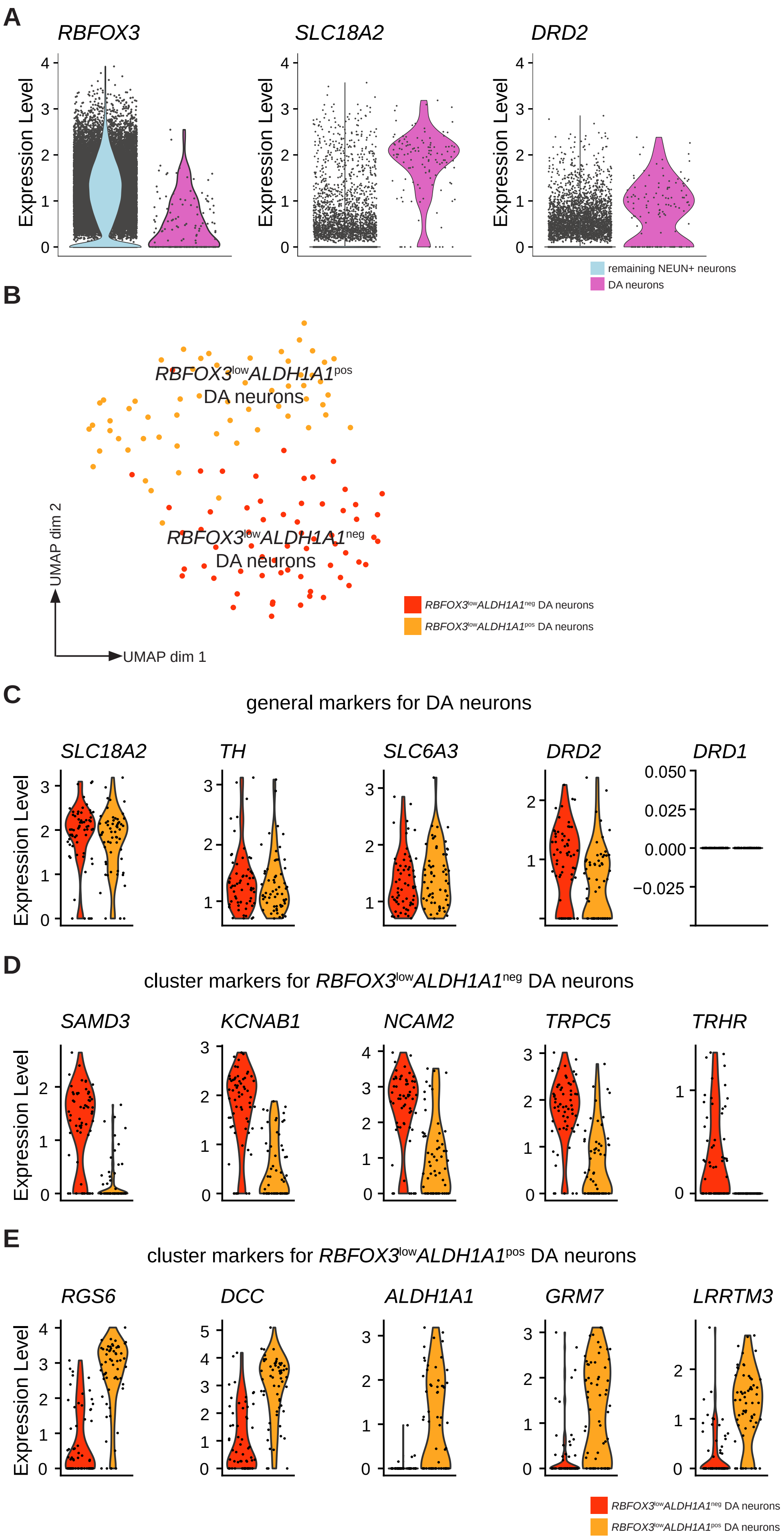

Supplementary Figure 4

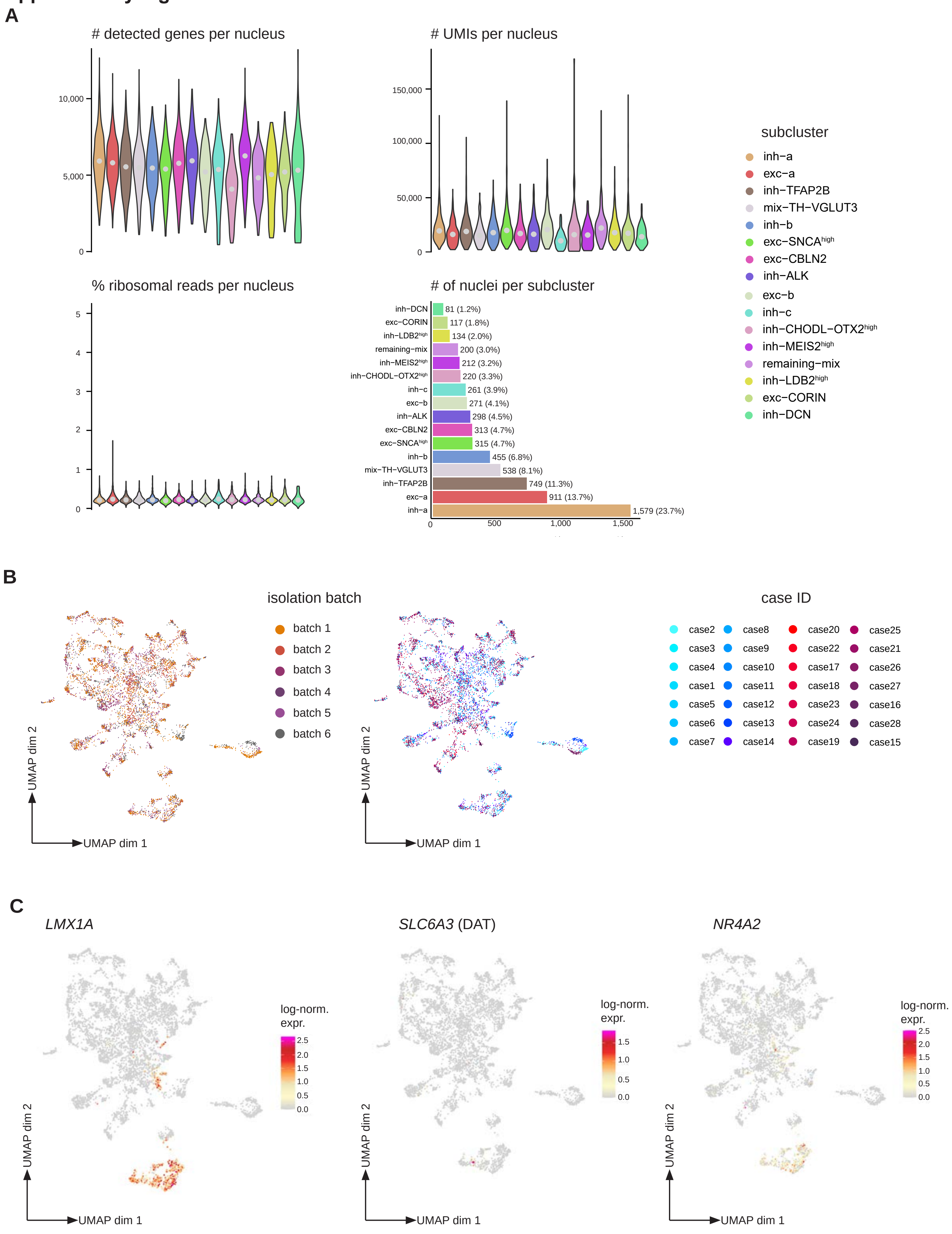

Supplementary Figure 5

A

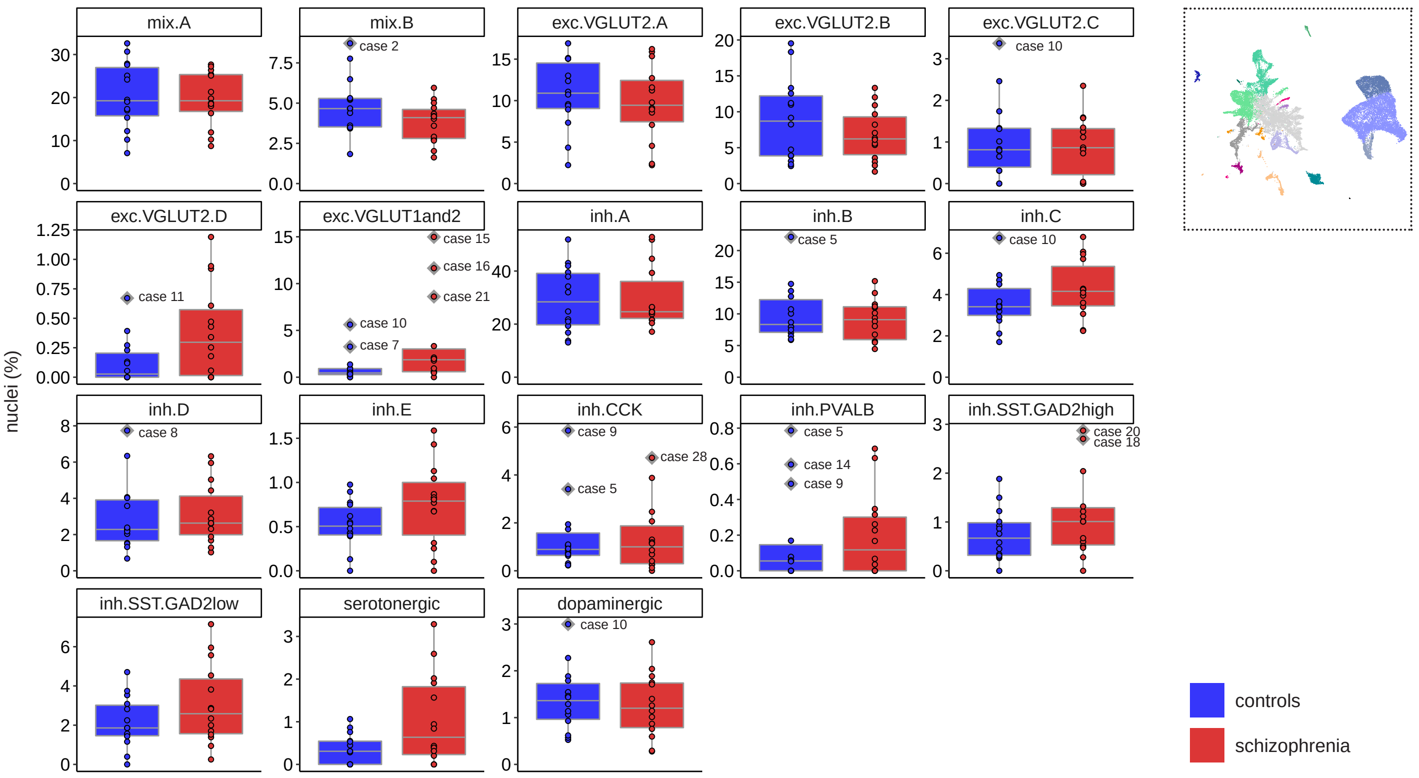

B

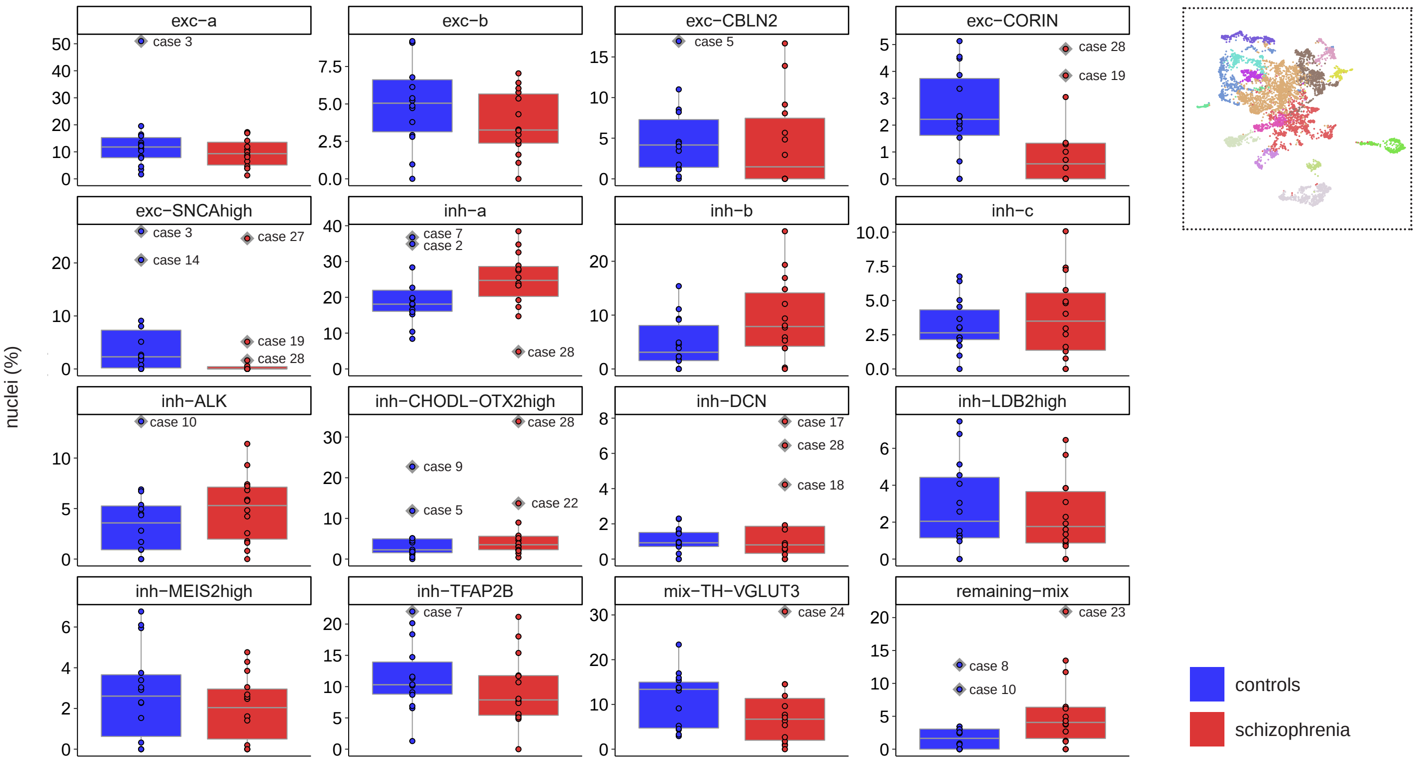

Supplementary Figure 6

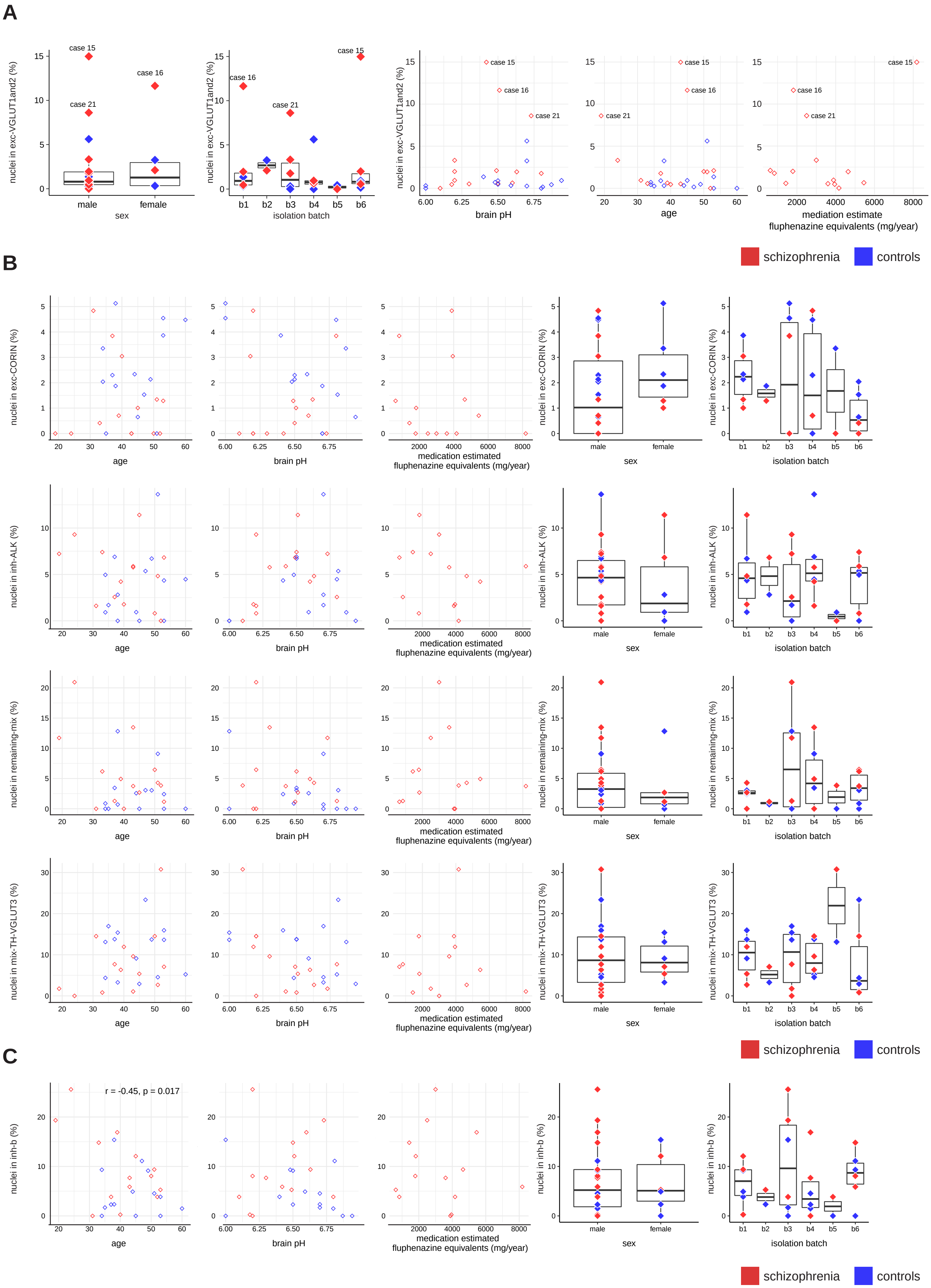

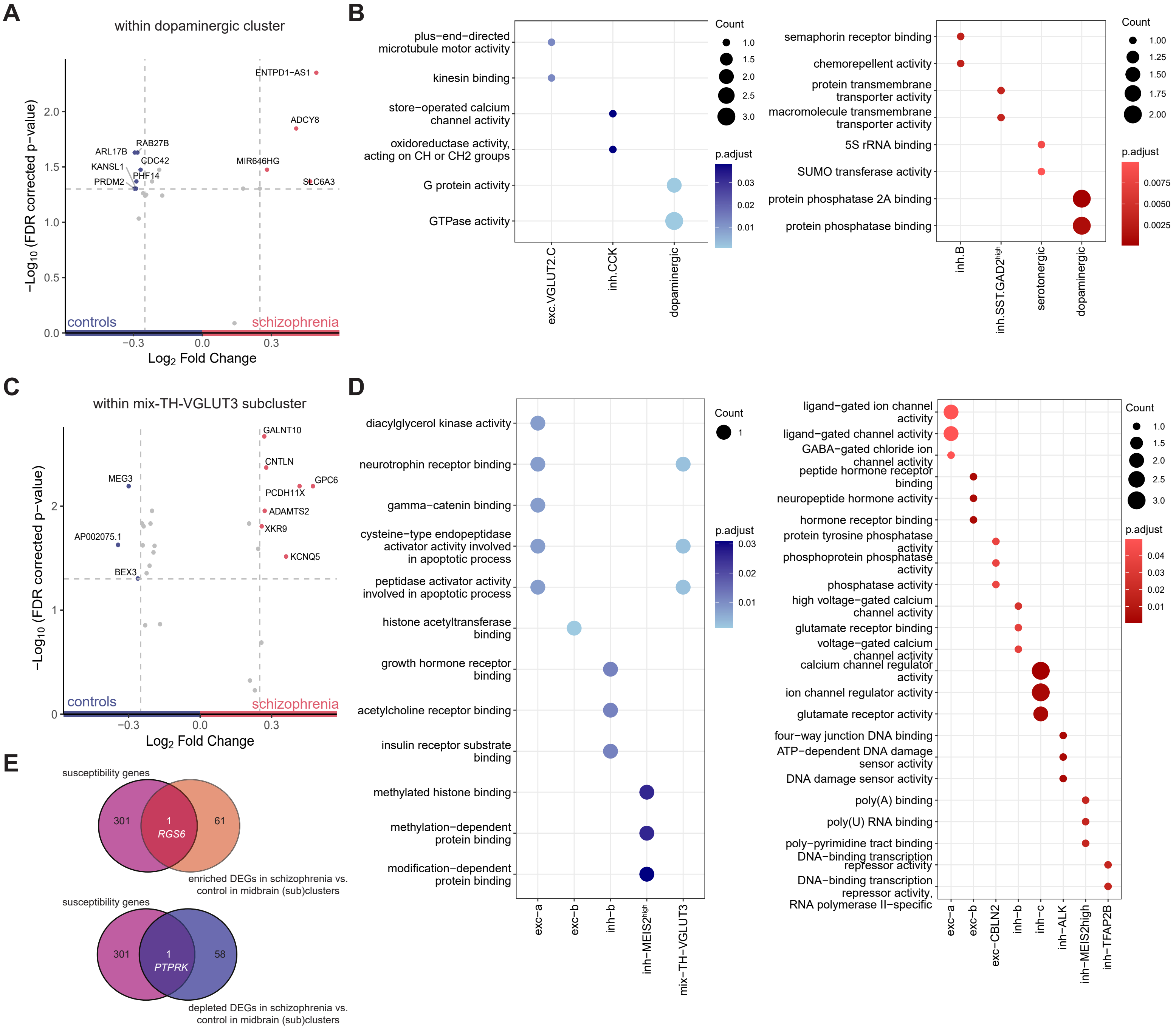
A
within dopaminergic cluster
−Log10 (FDR corrected p−value)
Log2 Fold Change
ENTPD1−AS1
ADCY8
RAB27B
ARL17B
CDC42
KANSL1
PHF14
PRDM2
MIR646HG
SLC6A3
controls
schizophrenia
B
plus−end−directed microtubule motor activity
kinesin binding
store−operated calcium channel activity
oxidoreductase activity, acting on CH or CH2 groups
G protein activity
GTPase activity
exc.VGLUT2.C
inh.CCK
dopaminergic
Count
p.adjust
semaphorin receptor binding
chemorepellent activity
protein transmembrane transporter activity
macromolecule transmembrane transporter activity
5S rRNA binding
SUMO transferase activity
protein phosphatase 2A binding
protein phosphatase binding
inh.B
inh.SST.GAD2high
serotonergic
dopaminergic
C
within mix-TH-VGLUT3 subcluster
GALNT10
CNTLN
GPC6
MEG3
PCDH11X
ADAMTS2
XKR9
AP002075.1
KCNQ5
BEX3
controls
schizophrenia
D
diacylglycerol kinase activity
neurotrophin receptor binding
gamma−catenin binding
cysteine−type endopeptidase activator activity involved in apoptotic process
peptidase activator activity involved in apoptotic process
histone acetyltransferase binding
growth hormone receptor binding
acetylcholine receptor binding
insulin receptor substrate binding
methylated histone binding
methylation−dependent protein binding
modification−dependent protein binding
exc−a
exc−b
inh−b
inh−MEIS2high
mix−TH−VGLUT3
ligand−gated ion channel activity
ligand−gated channel activity
GABA−gated chloride ion channel activity
peptide hormone receptor binding
neuropeptide hormone activity
hormone receptor binding
protein tyrosine phosphatase activity
phosphoprotein phosphatase activity
phosphatase activity
high voltage−gated calcium channel activity
glutamate receptor binding
voltage−gated calcium channel activity
calcium channel regulator activity
ion channel regulator activity
glutamate receptor activity
four−way junction DNA binding
ATP−dependent DNA damage sensor activity
DNA damage sensor activity
poly(A) binding
poly(U) RNA binding
poly−pyrimidine tract binding
DNA−binding transcription repressor activity
DNA−binding transcription repressor activity, RNA polymerase II−specific
exc−a
exc−b
exc−CBLN2
inh−b
inh−c
inh−ALK
inh−MEIS2high
inh−TFAP2B
E
susceptibility genes
301
1
RGS6
61
enriched DEGs in schizophrenia vs. control in midbrain (sub)clusters
susceptibility genes
301
1
PTPRK
58
depleted DEGs in schizophrenia vs. control in midbrain (sub)clusters
