# Supplementary material for: Schizophrenia-associated changes in neuronal subpopulations in the human midbrain
Source: Brain. 2024 Oct 14;148(4):1374–88. doi: 10.1093/brain/awae321 (PMC11969452; doi:10.1093/brain/awae321)
Supplement: awae321_Supplementary_Data [file awae321_supplementary_data.zip › brain-2023-02291-File006.pdf]

Supplementary Figure 1: snRNAseq quality control per cluster. A) Violin plots depicting the number of detected genes per nucleus (left), the number of unique molecular transcripts (UMIs) per nucleus (middle), and the % of ribosomal reads per nucleus (right). The white dot indicates the median. **B)** UMAPs depicting the individual expression of *GAD2* and *SLC17A6* (VGLUT2) and **C)** co-expression of *GAD2* and *SLC17A6*. **D)** Expression of selected neuronal and non-neuronal cell type markers per cluster. Size of the circles indicates the percentage of nuclei expressing the gene, colour indicates the average gene expression. **E)** UMAP as shown in Fig. 1B with the colours indicating nuclei isolation batch and individual cases. **F)** Stacked bar graph depicting cluster contribution per isolation batch and per individual case.

Supplementary Figure 2: Projection of identified clusters to an atlas of transcriptional neuronal diversity. **A**) UMAP representation of the supercluster annotations defined by Siletti et al.26 in 72,541 midbrain neurons from 3 control cases. **B**) Projection of neuronal clusters on Siletti et al.26 midbrain neurons visualized as prediction score. The prediction score (ranging from 0 to 1) represents the confidence that a given cell in the query dataset belongs to the predicted identity. **C**) The percentage of supercluster identities for each neuronal cluster. **D**) Violin plot depicting log-normalized expression level of ETV5, a cluster marker for inh-B. **E**) A schematic representation of the rostral midbrain at the level of the red nucleus and third cranial nerves (created with biorender.com). The lines indicate where the superior colliculus (SC) and cerebral peduncles (CP) were removed before analysis. The box indicates the periaqueductal grey and the third cranial nerve nucleus. The dashed box indicates the red nucleus. The dotted box indicates the substantia nigra. **F**) Representative images of immunohistochemical stainings for ETV5 (brown) and GFAP (black) in control midbrain (n=5) in the regions shown in **E**. ETV5 label in the substantia nigra is counterstained with Nissl. **G**) UMAPs depicting the co-expression of PVALB and SLC17A6, and co-expression of SLC17A6 (VGLUT2) and SLC17A7 (VGLUT1). **H**) Violin plot depicting log-normalized expression level of CYP26B1, a cluster marker for exc-VGLUT1and2. **I**) A representative image of immunohistochemical staining for CYP26B1 (brown, blue arrows) and GFAP (black) in control midbrain (n=5). Scale bar = 50µm.

Supplementary Figure 3: Subclustering of dopaminergic neurons in the midbrain. A) Violin plots depicting expression of *RBFOX3, SLC18A2* and *DRD2* in DA neurons (pink) and

in the remaining NEUN+ population (light blue). DA neurons were defined as nuclei co-expressing *TH* and *SLC6A3*. **B)** Unsupervised subclustering of 123 DA neurons (**Supplementary Table 5-6**). **C)** Violin plots depicting DA markers that were equally expressed in both DA subpopulations. **D-E)** Violin plots depicting expression of selected cluster marker genes ($log_2FC > 0.25$, *p*-adj. < 0.05) for the RBFOX3^{low}ALDH1A1^{neg} DA subpopulation and RBFOX3^{low}ALDH1A1^{pos} DA subpopulation (**Supplementary Table 6**).

Supplementary Figure 4: snRNAseq quality control per subcluster. A) Violin plots depicting quality control metrics per subpopulation (number of detected genes per nucleus, the number of UMIs per nucleus, and the % of ribosomal reads per nucleus). The white dot indicates the median. Bar plot depicting the total number of nuclei obtained per subcluster, with the percentage of the "mixed" population enclosed in brackets (**Supplementary Table 10**). **B)** UMAP as shown in Fig. 2A with the colours indicating isolation batch (left) and individual cases (right) **C)** UMAPs depicting the expression of DA markers *SLC6A3* (DAT), *LMX1A,* and *NR4A2.* Colour indicates log-normalized gene expression. Abbreviations: expr. = expression, log-norm. = log-normalized, UMAP = Uniform Manifold Approximation and Projection, UMIs = unique molecular identifiers

Supplementary Figure 5: Relative abundance of neuronal (sub)populations in schizophrenia and controls. A-B) Boxplots depicting the neuronal population (A) and subpopulation (B) proportions detected in schizophrenia and control cases (n = 14 per group). Each dot indicates a case, grey diamonds indicate outlier cases, and horizontal lines indicate the median. Outliers were defined as proportions outside of the third quartile plus 1.5 times the interquartile range. Group comparisons were carried out with a generalized linear model and *p*-values were corrected for multiple comparisons using the Benjamini-Hochberg procedure (**Supplementary Table 10, S12**).

Supplementary Figure 6: Relative abundance of neuronal (sub)populations and their relation to age, brain pH, medication estimate, sex, and isolation batch. A) Scatter or boxplot depicting the proportion of exc-VGLUT1and2 neurons detected per case on the vertical axis and the case-related variables brain pH, age, estimated medication usage, sex, and nuclear

isolation batch on the horizontal axis. **B)** Identical plots for neurons classified as "exc-CORIN", "inh-ALK", "remaining-mix", "mix-TH-VGLUT3" and **C)** "inh-b".

Supplementary Figure 7: Differentially expressed genes between schizophrenia and controls within neuronal (sub)populations and their associated GO terms. A) Volcano plot depicting differentially expressed genes (absolute log2 fold-change > 0.25, *p*-adjusted < 0.05) between schizophrenia and controls within the neuronal population "dopaminergic". The full list of DEGs for all populations is available in **Supplementary Table 13. B)** Significant GO terms that are associated with decreased (blue) and increased (red) gene expression changes in schizophrenia cases compared to controls per population. If a population is absent this indicates no significantly enriched GO term was detected. **C)** Volcano plot depicting differentially expressed genes (absolute log2 fold-change > 0.25, *p*-adjusted < 0.05) between schizophrenia and controls within the neuronal subpopulation "mix-TH-VGLUT3". The full list of DEGs is available in **Supplementary Table 14**. **D)** Significant GO terms that are associated with decreased (blue) and increased (red) gene expression changes in schizophrenia cases compared to controls per subpopulation. If a subpopulation is absent on the horizontal axis this indicates no significant GO term was detected. Abbreviations: *p*.adjust = FDR-adjusted *p*-value, GO = gene ontology, count = number of genes. **E)** Overlap between schizophrenia susceptibility genes (**Supplementary Table 15**) and differentially expressed genes in schizophrenia versus control in all clusters and subclusters (**Supplementary Table 13-14**).

References

26. Siletti K, Hodge R, Mossi Albiach A, et al. Transcriptomic diversity of cell types across the adult human brain. *Science*. 2023;382(6667):2022.10.12.511898. doi:10.1126/science.add7046

27. Wang D, Liu S, Warrell J, et al. Comprehensive functional genomic resource and integrative model for the human brain. *Science*. 2018;362(6420). doi:10.1126/science.aat8464
